# Simulating CD8 T cell exhaustion: A comprehensive approach

**DOI:** 10.1016/j.isci.2025.112897

**Published:** 2025-06-12

**Authors:** Andrea J. Manrique-Rincón, Ben Foster, Stuart Horswell, David A. Goulding, David J. Adams, Anneliese O. Speak

**Affiliations:** 1 Cambridge Institute of Therapeutic Immunology and Infectious Disease, University of Cambridge, Cambridge, UK; 2 Open Targets, Wellcome Genome Campus, Hinxton, Cambridge, UK; 3 Wellcome Sanger Institute, Hinxton, Cambridge, UK

**Keywords:** Immunology, Components of the immune system

## Abstract

Immunotherapy has transformed cancer treatment but benefits only some patients, and predictive biomarkers are lacking. One correlate of response is the reinvigoration of a subset of CD8 T cells that have an exhausted phenotype and impaired functionality. To develop effective therapies, reproducible models are required to identify candidate target genes that enable reversal of T cell exhaustion. Here, we describe an *in vitro* model by chronically stimulating T cells with their cognate antigen, followed by temporal phenotypic characterization. This model recapitulates many critical hallmarks of exhaustion, including expression of canonical surface markers, impaired proliferation, reduced cytokine production, decreased cytotoxic granule release, and metabolic alterations. Two *in vivo* models validate these results and establish a gene signature shared by *in vitro* and *in vivo* exhausted states. Critically, this signature is observed in tumor infiltrating T cells from multiple human tumor types, validating the translational potential of this model for discovering therapies.

## Highlights

• Reproducible *in vitro* model of T cell exhaustion developed
• Exhausted T cell phenotype systematically characterized over time
• Model benchmarked against *in vivo* samples to confirm relevance
• Identification of core shared gene signature between mouse and human

## Graphical abstract

**Figure:**
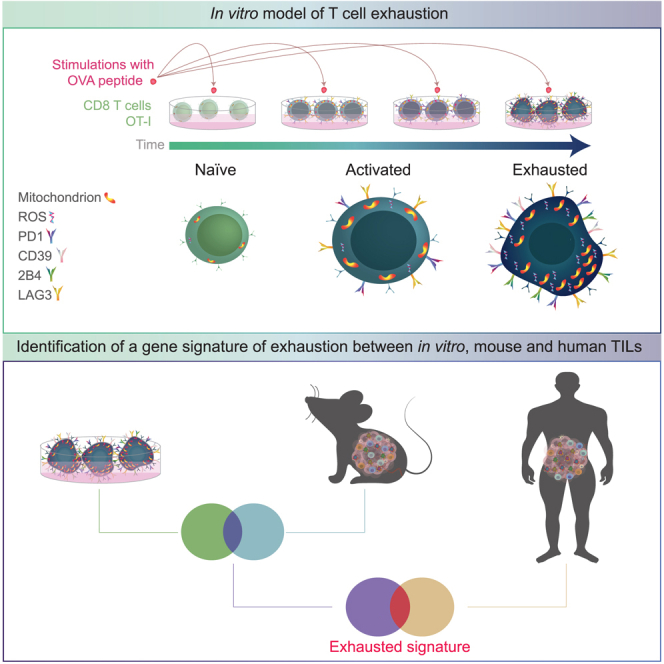

## Introduction

Exhausted T cells emerge when their antigen remains present for long periods, impairing immune responses including against viral infections and cancer.^1^ These cells were initially described after mice were infected with lymphocytic choriomeningitis virus (LCMV) clone 13 strain, which results in a chronic persistent infection.^2^^,^^3^^,^^4^ Exhausted T cells are characterized by an impairment in functionality, proliferation, and metabolic alterations. This is accompanied by high expression of inhibitory receptors (IR) including programmed cell death protein 1 (PD1), cytotoxic T-lymphocyte association protein 4 (CTLA4), T cell immunoglobulin and mucin domain-containing protein 3 (TIM3), lymphocyte activation gene-3 (LAG3), CD39, CD244 (2B4), and TIGIT.^5^ These changes can be identified at the transcript level and via epigenetic alterations.^6^

While it is evident that tumor antigen reactive T cells are present within most tumors, their ability to eliminate cancer cells is limited. This is because the tumor microenvironment (TME) provides several conditions besides chronic antigen exposure to maintain the exhausted phenotype of T cells, including hypoxia,^7^^,^^8^^,^^9^ lack of nutrients,^10^^,^^11^ immunosuppressive cells such as regulatory T cells^12^ and Myeloid-derived suppressor cells (MDSC),^6^ and the expression of inhibitory ligands, for example, PD-L1, and PD-L2.^13^^,^^14^^,^^15^ These signals trigger and maintain tumor reactive T cells in an exhausted state exemplified by decreased proliferative capacity, reduced effector function, expression of IRs and metabolic changes including increased ROS and mitochondria mass with a decrease in oxygen consumption.^1^^,^^16^^,^^17^^,^^18^

The success of therapies that boost the immune system to respond against cancer cells (immunotherapies) has brought a revolution characterized by a drive to identify ways to manipulate different components of the immune response in order to avoid immune escape, and ultimately to eradicate tumors. However, an essential factor that hinders this potential is the exhausted phenotype of tumor-reactive cells.^19^^,^^20^ One of the most successful immunotherapies in clinical use is immune checkpoint blockade (ICB). This therapy disrupts the interaction between IRs on exhausted cells and their ligands.^21^^,^^22^^,^^23^ However, only a fraction of patients will respond and many will suffer significant adverse events. Despite multiple clinical studies, biomarkers to predict patient response or risk of adverse events to ICB have not been fully characterized. Temporal investigations to identify the kinetics and identity of the cells that respond to ICB have shown that only a subset of exhausted cells improve their functionality after ICB administration.^24^^,^^25^^,^^26^^,^^27^ Furthermore, ICB does not reverse the epigenetic changes that occur in the exhausted T cells and thus cells can revert after treatment cessation. In addition to ICB, another major type of immunotherapy is adoptive transfer of tumor reactive cells, in particular engineered chimeric antigen receptors T cells (CAR-T). CAR-T cells have shown remarkable responses in many hematological malignancies, yet their application to the treatment of solid tumors has been hindered by the suppressive tumor microenvironment which rapidly converts the cells into an exhausted state.^28^^,^^29^^,^^30^^,^^31^ With the advances in cellular manufacturing it could be possible to engineer exhaustion resistant CAR-T if suitable genetic targets were identified.

To better understand how to improve the reactive capacity of exhausted cells and harness it for immunotherapy, it is necessary to construct robust and reliable models of T cell exhaustion with translational potential. Here, we develop an *in vitro* model that uses exposure to their cognate antigen, mimicking chronic antigen exposure. This *in vitro* model reproduces the main characteristics of exhausted T cells and was validated using cells isolated from tumor-infiltrating lymphocytes (TILs). RNA-seq of the *in vitro* and *in vivo* cells identifies shared gene signatures specifically associated with exhausted T cells. To confirm the translational potential of this model, we compared the shared exhaustion signature we have identified from our models to a human pan-cancer T cell atlas^32^ generating a list of 259 genes that are common between the models which includes 25 transcription factors.

## Results

### A model of exhaustion *in vitro* that recapitulates the main hallmarks of exhaustion upon chronic stimulation

**Figure 1:**
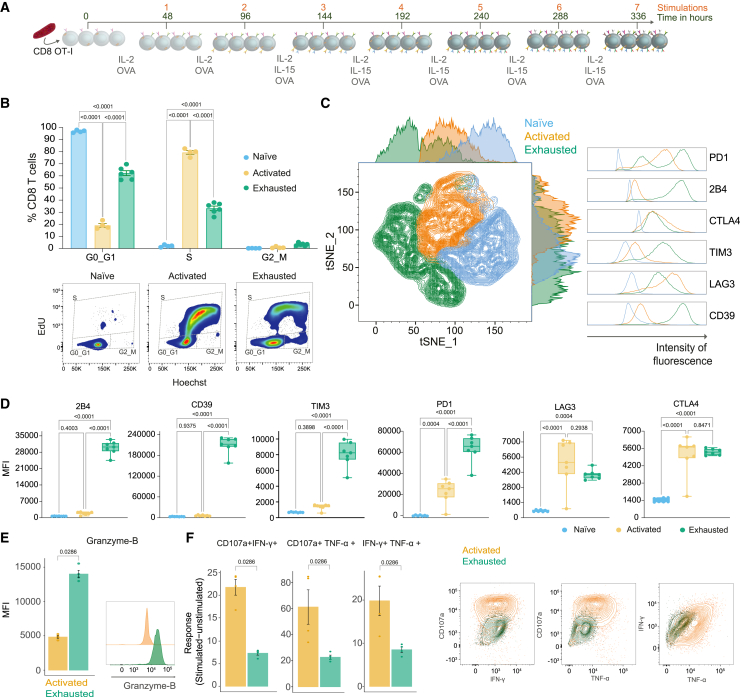
Characterization of the development of *in vitro* exhausted CD8 T cells Freshly isolated cells (Naive), cells activated with SIINFEKL for 24h (Activated), and chronically stimulated cells (Exhausted) were phenotypically characterized. (A) *In vitro* experimental design of chronic stimulation to produce exhausted T cells. (B) Representative gating strategy and percentages of T cells per cell-cycle stage obtained by EdU and Hoescht staining. (C) tSNE using IRs with representative histograms for indicated markers on naive, activated, and *in vitro* exhausted T cells. (D) Quantification of geometric mean fluorescence intensity (MFI) of individual markers. (E) Intracellular expression of Granzyme B with representative histograms. (F) Background unstimulated corrected percentage of double-positive populations of CD107a+ IFNγ+, CD107a+ TNFα+, and IFNγ+ TNFα+ in stimulated cells with representative contour plot of stimulated samples. Representative data from three independent experiments. Mean ± SEM shown with symbols representing individual mice. Statistical analysis performed by two-way ANOVA with a Tukey’s multiple-comparisons test (B and D) or Mann–Whitney tests (E and F). Only significant *p* values indicated in the figure.

Exhausted T cells are produced via chronic stimulation through the T cell receptor (TCR) with cognate antigen. To recapitulate this process *in vitro*, we used cells from OT-I mice carrying a transgenic TCR that recognizes the H-2K(b)/SIINFEKL OVA (257–264) epitope.^33^ Freshly isolated CD8 T cells from spleens of OT-I mice were stimulated every 48 hours in the presence of IL-2 and after the second re-stimulation were also supplemented with IL-15 (Figure 1A). After seven stimulations, these cells were analyzed in comparison to early activated T cells (24 hours with SIINFEKL), and to freshly isolated naive T cells. The *in vitro* exhausted cells showed a reduced ability to proliferate, compared to activated T cells, as evidenced by a reduction in cells in the S phase, determined by EdU incorporation (Figure 1B). The expression of hallmark IRs including 2B4, CD39, TIM3, PD1, LAG3, and CTLA4 was assessed and these markers were highly expressed in exhausted T cells (Figures 1C and 1D). TIM3, CD39, and 2B4 were particularly highly expressed on exhausted cells relative to activated cells enabling their use to distinguish these populations. Notably other frequently used exhaustion markers such as PD1, LAG3, and CTLA4 were also highly expressed by activated cells and in the case of LAG3 there was not a significant difference between activated and exhausted (Figures 1C and 1D). Another critical hallmark of exhausted T cells is their reduced functional capacity regarding cytokine production and cytotoxicity. In comparison with cells activated for 24 hours and expanded for 72 hours the *in vitro* exhausted cells contained higher levels of Granzyme B, a key component of cytotoxic granules, consistent with the phenotype reported in CD8 TILs^24^ (Figure 1E). The functional impairment of the *in vitro* exhausted cells was evidenced by the reduced polyfunctionality of the cells after restimulation with SIINFEKL, either the ability to degranulate and produce cytokines (CD107a+ IFNγ+ or CD107a+ TNFα+) or to produce two cytokines simultaneously (IFNγ+ TNFα+) (Figure 1F). Looking at the pool of exhausted cells and compared to the four-cell-stage developmental framework of exhaustion,^34^ defined by Ly108 and CD69, these *in vitro* exhausted T cells lie between intermediately and terminally exhausted (Figure S1). In order to define the role of the tumor microenvironment in the development of these features and to provide a benchmark we compared the *in vitro* generated cells to those isolated from two different syngeneic orthotopic models, which have different levels of immune infiltration and response to immunotherapy. B16-F10 (melanoma, “cold”) and MC38 (colorectal carcinoma, “hot”) were modified to stably express ovalbumin after genome integration using the PiggyBac transposon system. After subcutaneous implantation, tumors were allowed to grow for nine days to enable sufficient time for the tumor microenvironment to develop prior to collection of the tumor, draining lymph node, and spleen. As expected, there was a significant increase in PD1 expression within the B16-F10-OVA and MC38-OVA tumors compared to lymph nodes and spleen, with MC38-OVA CD8 T cells expressing higher PD1 relative to B16-F10-OVA (Figure S2). Also, there was a significant increase in CD39 and Granzyme B expression on CD8 from MC38-OVA tumors relative to lymph node and spleen. B16-F10-OVA tumors did not have significantly elevated levels of CD39 or Granzyme B compared to lymph node and spleen and actually MC38-OVA tumors were significantly higher than B16-F10-OVA. Notably the *in vitro* exhausted cells had a considerably higher expression of PD1, CD39, and Granzyme B compared to those from the tumors, likely due to timepoint that was assessed not enabling the full development of an exhausted phenotype *in vivo* and comparing to a bulk population of CD8 T cells where only a subset of CD8 will have a TCR that can detect tumor antigens. LAG3 was not significantly elevated in the tumors relative to the lymph node or spleen similar to that observed between *in vitro* activated and exhausted.

### *In vitro* exhausted cells have an altered metabolism and higher numbers of mitochondria

**Figure 2:**
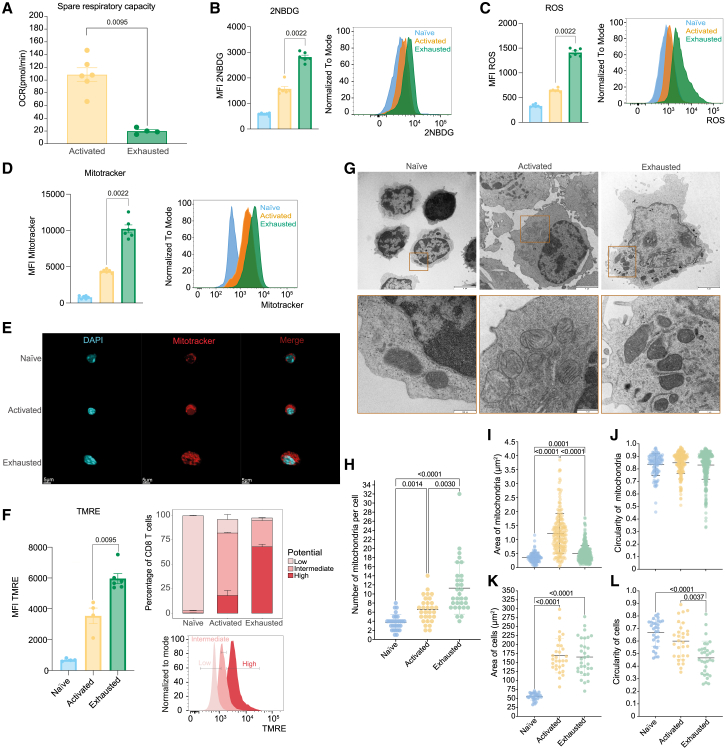
Metabolic alterations of *in vitro* exhausted T cells Freshly isolated cells (Naive), cells activated with SIINFEKL for 24h (Activated), and chronically stimulated cells (Exhausted) characterized for metabolic changes. (A) Quantification of the spare respiratory capacity. (B) Glucose uptake quantification using 2-NBDG, representative histograms are shown. (C) Determination of cellular ROS content with representative histograms shown. (D) Measurement of mitochondrial mass with MitoTracker Deep Red, with representative histograms shown. (E) Confocal visualization of mitochondria, 3D view with MitoTracker in red and DAPI for nucleus in blue. Scale bar = 5 μm. (F) Mitochondrial membrane potential assessed using TMRE, representative histograms with gates of potential. (G) Transmission electron microscopy (TEM) of naive, activated, and exhausted T cells with inset zoom on mitochondria, scale bars are as follows for cells and mitochondria images; naive 4 μm and 500 nm; activated 4 μm and 1 μm and exhausted 4 μm and 1 μm. (H) Number of mitochondria per cell. (I–L) Area and circularity of mitochondria and cells respectively. Representative data from three (A-E) and two (F) independent experiments. For TEM at least three sample preparations per group were imaged and at least 27 cells per sample were quantified. Mean and ±SEM shown with symbols representing individual mice (A-F), individual cells (H, K, and L) or individual mitochondria (I and J). Statistical analyses were performed by Mann–Whitney tests (A-D and F) and Kruskal-Wallis ANOVA with Dunn’s multiple comparisons test (H-L). Only significant *p* values indicated in the figure.

There is a change in the metabolism of T cells after the first encounter with an antigen. This event triggers a conversion from mitochondria-dependent oxidative phosphorylation (OXPHOS) to aerobic glycolysis, and once the effector stage has passed, memory cells return to a more quiescent state dictated by OXPHOS.^17^ Chronic stimulation leads to disruption in the metabolism of exhausted compared to activated cells in multiple ways. The spare respiratory capacity (SRC) is a key factor in determining a cell’s response to stress and has previously been shown to vary between different CD8 subsets with a low SRC in effector cells and high SRC in memory. We determined the SRC of the *in vitro* exhausted T cells compared to activated and observed a significant reduction in the SRC of exhausted cells (Figure 2A) accompanied by a reduced extracellular acidification rate (ECAR) (Figure S3) Exhausted cells showed increased glucose uptake (Figure 2B) with higher levels of oxygen species (ROS) (Figure 2C). The exhausted cells had increased mitochondrial mass, as determined by MitoTracker (Figures 2D and 2E), and an increase in the mitochondrial membrane potential measured with TMRE (Figure 2F). In order to quantify mitochondrial number and morphology at an ultrastructural resolution we performed transmission electron microscopy (TEM) (Figure 2G). Mitochondrial content of activated CD8 T cells is twice that observed in naive CD8 T cells, with exhausted cells having a further 2-fold increase compared to activated cells (Figures 2G and 2H). However, the mitochondria of activated cells are bigger than those in exhausted and naive cells (Figure 2I) with similar circularity (Figure 2J). While both activated and exhausted cells are larger than naive by 3.5-fold (Figure 2K), the exhausted cells showed a reduction in their circularity and a more irregular shape (Figures 2G and 2L).

### Temporal acquisition of exhaustion phenotypes

**Figure 3:**
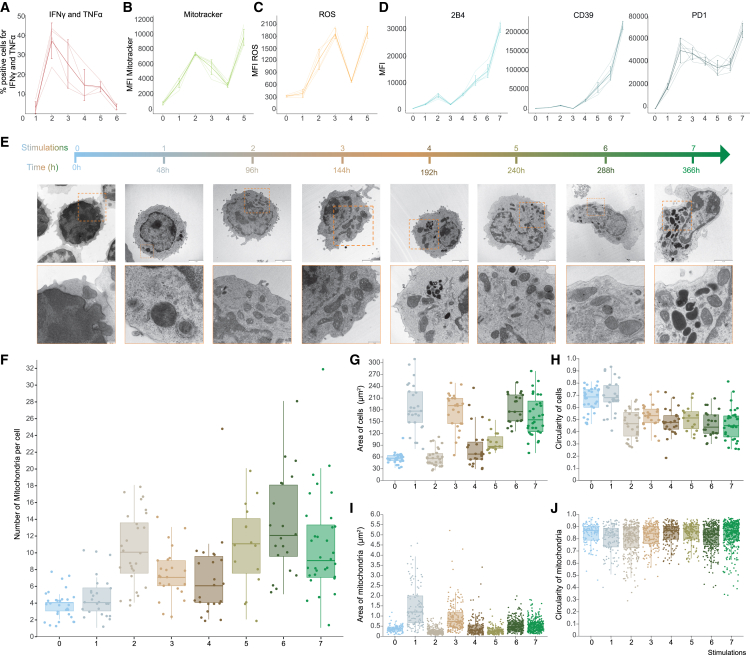
Time course of phenotypic exhaustion development (A) Percentage of cells double-positive for IFNγ and TNFα after each stimulation. (B) Mitochondrial mass as determined by MitoTracker staining. (C) Cellular ROS content. (D) Expression of 2B4, CD39 and PD1 after each stimulation. (E) Representative TEM images of cells and inset zoom of region for mitochondria, scale bars are as follows; naive 3 μm and 500 nm; 48h 4 μm and 500 nm; 96h 3 μm and 500 nm; 144h 3 μm and 1 μm; 192h 3 μm and 1 μm; 240h 3 μm and 500 nm; 288h 4 μm and 500 nm; 366h 3 μm and 500 nm. (F) Number of mitochondria per cell. (G) Area of cells. (H) Circularity of cells. (I) Area of mitochondria. (J) Circularity of mitochondria. Representative data from two (A-D) independent experiments, light lines individual mice and dark lines mean ± SD with *n* = 7 mice per measurement. For TEM at least 3 sample preparations per group were imaged and 27 cells per sample were quantified. Boxplots shown at median with interquartile range with symbols representing individual cells (F, G, and H) or individual mitochondria (I and J). Statistical analysis was performed with a Brown-Forsythe and Welch ANOVA test with multiple comparisons for (F–J) with all *p* values in Table S1.

To better understand the order in which these alterations occurred we conducted a time-course analysis, collecting cells after each stimulation. We quantified the progression of exhaustion by determining polyfunctional cytokine production after stimulation (IFNγ and TNFα double-positive cells) (Figure 3A). These results showed that after three stimulations the functionality of the cells decreases, and polyfunctional cytokine producing cells are virtually undetectable after the sixth stimulation. Mitochondrial mass increased and peaked initially after the second stimulation in line with the maximal cytokine polyfunctionality, an indicator of the peak effector phase. There was then a decrease in mitochondrial mass before a final increase toward the fifth stimulation as the cells entered the dysfunctional state (Figure 3B). ROS followed a similar trend to mitochondrial mass although the initial peak was after the third stimulation before a decrease and final terminal increase at the fifth stimulation (Figure 3C). While high surface expression of 2B4 and CD39 was specifically observed at the later timepoints, PD1 remained highly expressed throughout the time course after activation (Figure 3D). The development of exhausted T cells was accompanied by a decrease in viability (Figure S4).

We used the time course to evaluate the morphology and features of the cells and their mitochondria using TEM (Figure 3E). We collected samples after each stimulation and evaluated size, area, number, and circularity at both cellular and mitochondrial level (Figures 3F–3J). This analogous method of determining mitochondrial mass showed the same trend as was observed using MitoTracker staining and quantification by flow cytometry (Figures 2D, 2E, 3B, and 3F). The area of the cells had an initial cyclical expansion and contraction before maintaining an area of approximately three times that of unstimulated cells (Figure 3G), whilst the circularity of the cells decreased from the second stimulation and remained at this reduced level for the duration of the experiment (Figure 3H). The area of the mitochondria initially followed a similar recurring expansion and contraction aligned with the area of the cells, however, after the fifth stimulation there was no correlation and the mitochondrial area was relatively constant (Figure 3I). There was no noticeable difference in the circularity of the mitochondria at any timepoint (Figure 3J). These results illustrate that the phenotypic, functional and metabolic alterations observed during T cell exhaustion show marked differences in their temporal acquisition during chronic antigen stimulation.

### *In vivo* model of T cell exhaustion in response to cancer

**Figure 4:**
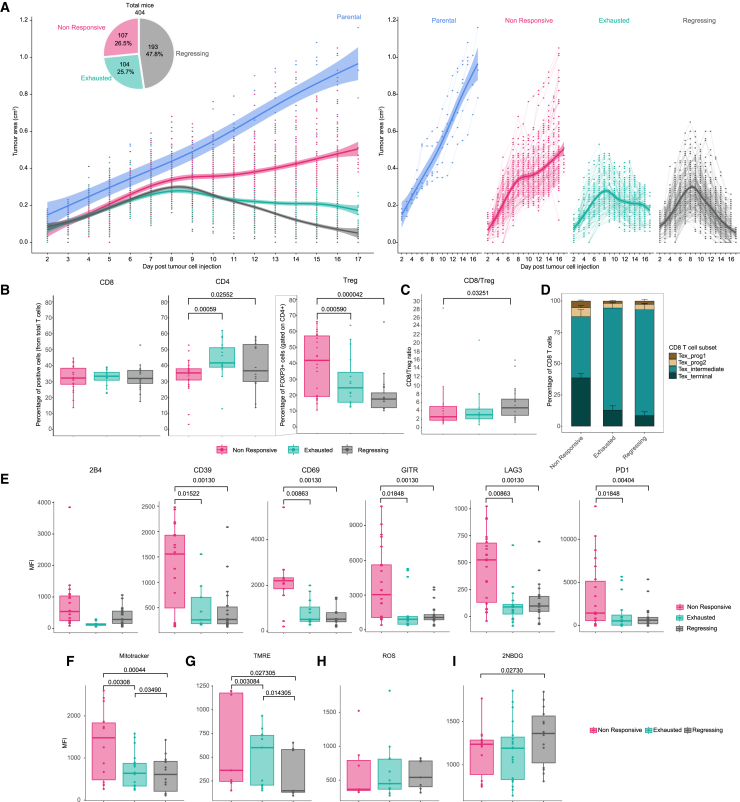
Characterization of an *in vivo* exhaustion model (A) Tumor growth of MC38 tumors in mice after injection of MC38 parental (blue) or MC38-OVA with three different responses as determined by INSPECTumours (non-responsive in pink, exhausted in green, and regressing in gray). Pie chart with percentages and number of individuals per response. Individual graphs per response show average trend line in bold with 95% confidence interval with individual mice indicated by points and fine lines. (B) Percentage of T cells subsets within tumors, CD4 and CD8 are expressed as percentage of alive cells and Tregs as percentage of CD4^+^ viable cells. (C) Ratio of CD8/Treg in tumors. (D) Relative proportions of exhausted subsets in CD8s TILs. (E) MFI of exhaustion markers on CD8 TILs. (F) Mitochondrial mass of CD8s TILs determined by MFI of MitoTracker staining. (G) Mitochondrial potential of CD8s TILs determined by MFI of TMRE. (H) MFI of cellular ROS of CD8s TILs. (I) Glucose update in CD8 TILs as assessed by MFI of 2-NBDG. Representative data from 10 pooled experiments (A), four pooled experiments (B–E), and two pooled experiments (F–I). Boxplots show mean and interquartile range, symbols represent individual mice. Mean ± SEM is shown (E, G) with symbols representing individual mice. Statistical analyses (B-I) were performed using PhenStat with time as a fixed-effect model and after adjusting for multiple testing with the Benjamini & Hochberg method. Only significant *p* values are indicated in the figures.

In our *in vivo* model, when we compared the tumor growth of the MC38-OVA cell line stably expressing ovalbumin with the parental, the ovalbumin expressing tumors grew at a slower rate compared to the parental (MC38) in immunocompetent mice (Figure 4A). After day nine, it was noticeable that around two-thirds of the tumors began to show a response with the tumors getting smaller in the absence of any treatment whereas others continued to grow. With more time approximately one-third of these tumors resumed growing. The emergence of three distinct responses was unexpected given the same tumor cell line was injected. To further validate this observation, we repeated the experiment in different WT colonies and in another animal facility observing the same effect in each replicate. We adapted a recently reported Bayesian model^35^ to categorize the responses into the three groups. As there is no treatment applied and because the tumors grew slower than the parental MC38 cells line we used the top quartile (100 mice) on the basis of the area under the curve for tumor growth to define the non-linear model. Using the 75% bounds of the model to define the individual points and requiring at least two data points from day 10 to be outside the control range for the tumor, including the final measurement, to be classified as a “responder” otherwise they were “non-responsive”. The “responders” were further subdivided into those with a negative growth rate from day 10, with a growth rate *p* value <0.05 (as determined by a t-test) were classed as “regressing” tumors and the rest as ‘exhausted’. The graph shown in Figure 4A is the result of 10 pooled experiments that were independent of age, cage, and sex (Table S2). These patterns of tumor responses appeared in similar proportions in each experiment, with around half (47.8%) of the animals presenting complete regression and the other half being divided between exhausted (25.7%) and non-responsive (26.5%) (Figure 4A).

Considering that together with exhausted CD8 T cells, CD4 T cell subsets are the key T cell populations within the TME with implications in prognosis, we decided to look at their infiltration in the three responses. MC38-OVA cells were orthotopically implanted into FOXP3_IRES_mRFP (FIR) mice^36^ enabling direct *ex vivo* identification without fixation and permeabilization. Immunophenotyping of tumors at 15 days post injection, showed differences in the fraction of infiltrating CD4 T cells and the percentage of CD4 T cells that were Tregs. Regressing and exhausted tumors contained significantly more CD4 cells compared to non-responsive tumors, which had a higher fraction of CD4s that were Tregs (Figure 4B). The CD8/Treg ratio was higher for regressing than non-responsive (Figure 4C). No differences were observed in draining lymph nodes or spleen (Figure S5).

Next, we determined the expression of key exhaustion related phenotypic markers that were altered in our *in vitro* model and observed similar changes in the *in vivo* model. The frequency of exhausted CD8 subsets, defined by CD69 and Ly108, was determined and the terminally exhausted subset was higher in the non-responsive and the intermediately exhausted higher on the regressing (Figure 4D). The expression of CD39, CD69, GITR, LAG3 and PD1 was significantly higher on the cells from non-responsive tumors compared to both exhausted and regressing tumors (Figure 4E). These changes were unique to the tumor as they were not observed in the lymph node or spleen (Figure S5).

Mitochondrial mass and mitochondrial potential (measured by MitoTracker Deep Red and TMRE, respectively) was higher in non-responsive tumors, followed by exhausted and then regressing (Figures 4F and 4G). Total ROS was the same in all responses (Figure 4H) with higher uptake of glucose in the T cells from the regressing compared to non-responsive (Figure 4I). The development of this *in vivo* model with different immune responses provides a unique opportunity to study immune escape, and recurrence, and could be used to analyze other populations involved in antitumor immune response.

### Transcriptomics signatures of exhaustion *in vitro* and *in vivo*

To understand the influence of the microenvironment in the development of T cell exhaustion we performed adoptive transfer of *in vitro* activated OT-I T cells into mice bearing exhausted tumors. Mice were subcutaneously implanted with MC38-OVA cells and after 16 days a cohort of 42 mice with tumors that initially showed some response were adoptively transferred with OT-I cells after 24 hours of *in vitro* activation. These mice were monitored for tumor growth and a subset of three mice identified which had a delayed tumor outgrowth (Figure S6A). Following 21 days of *in vivo* selection we collected tumors and draining lymph nodes for analysis. We isolated the CD8 T cells, and compared the expression of characteristic exhaustion markers (PD1, 2B4, CD39, and CTLA4) from host and transferred CD8s (Figure S6B). There was no significant difference between the host and transferred CD8 T cell expression of the markers although for CD39, 2B4, and CTLA4 there was a trend for higher levels on the transferred CD8, perhaps due to the presence of the OVA protein within the tumor.

**Figure 5:**
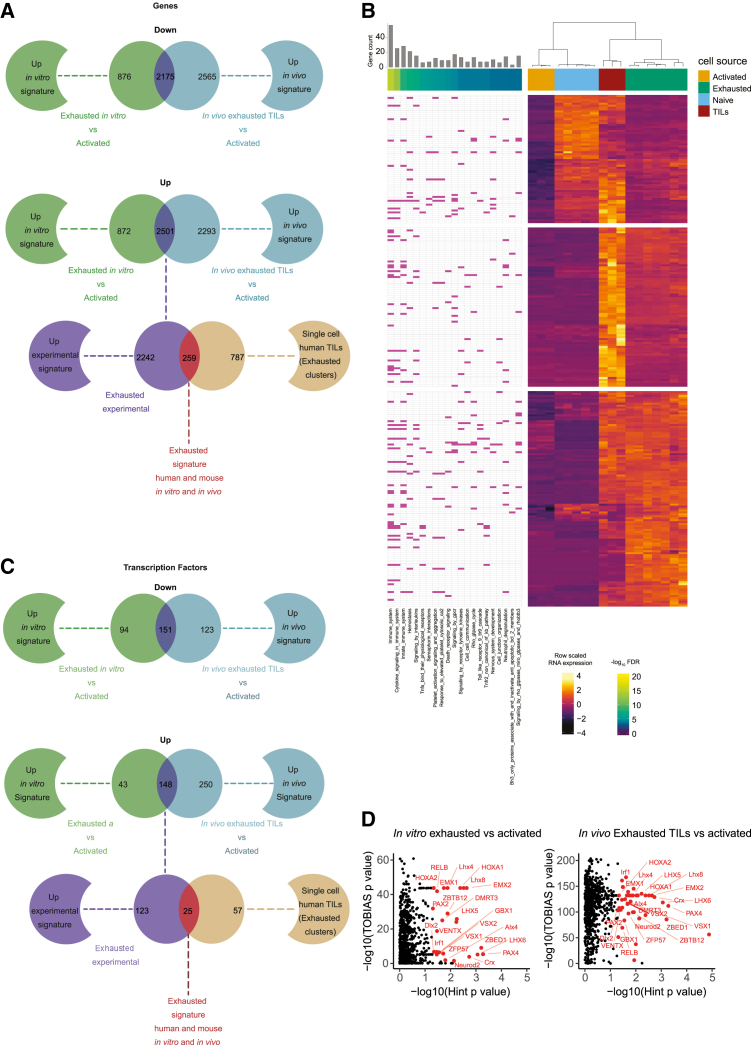
Signatures of exhaustion (A) Venn diagram of differentially down or up regulated genes in the indicated comparisons. (B) Heatmap of the conserved interspecies core exhausted genes showing expression of naive, activated, and exhausted *in vitro* CD8 and *in vivo* CD8 TILs for the exhausted signature. Enriched Reactome pathways for genes from GSEA indicated, with FDR *q*-value for enrichment and number of genes per pathway. (C) Venn diagram of differentially down or up regulated transcription factors in the indicated groups. (D) Enriched transcription factor motifs defined by TOBIAS and Hint. Red dots indicate motifs that are significant *p* < 0.05 for both methods and annotations are for motifs present both *in vitro* (left) and *in vivo* (right).

To fully investigate the impact of the tumor microenvironment we performed bulk RNA-seq on the sorted CD8 TILs from these exhausted tumors and the *in vitro* T cells (naive, activated and exhausted). First we identified the overlapping differentially expressed genes (DEGs) that were present in the *in vitro* exhausted T cells and the *in vivo* exhausted T cells, in comparison to activated T cells, to generate the *exhausted experimental signature* consisting of 2175 down regulated genes and 2501 up regulated genes (Figure 5A). Next we compared these enriched DEGs to those identified from scRNA-seq analysis of human TILs isolated from multiple cancer types^32^ with a focus on those in the annotated CD8 exhausted clusters to generate *the exhausted signature* (Figure 5A). This yielded 259 genes that are conserved between human and mouse and are present in both the *in vitro* and *in vivo* murine models representing key targets for therapeutic intervention (Table S3). All the comparisons were performed against early activated CD8 T cells to eliminate genes that are also critical for T cell activation. Three distinct clusters of expression patterns were identified (Figure 5B). Analysis of Reactome pathways via GSEA^37^ demonstrated significant enrichment of immune related pathways in these 259 shared mouse and human genes (Figures 5B and S7; Table S3). In contrast down regulated genes were enriched in pathways linked to cell cycle, telomeres, chromosomal stability and DNA repair (Figure S7; Table S3). In addition to many known exhaustion genes, including *Havcr2*, *Entpd1*, and *Pdcd1,* there are numerous genes within this signature that represent unexplored candidates for follow up studies with no previous link to T cell biology. Given the critical role of transcription factors in the development and establishment of exhaustion,^6^^,^^38^^,^^39^ we filtered for transcription factors within the groups and signatures which identified 25 upregulated transcription factor genes between the groups (Figures 5C and S8; Table S4). Of particular interest these genes included *Bhlhe40* and *Rbpj* which have both been recently identified as key transcription factors for CD8 T cell exhaustion by CRISPR screens.^40^^,^^41^

Epigenetic changes have previously been reported in exhausted T cells.^6^^,^^34^^,^^42^^,^^43^ To further validate our models and to identify any potentially epigenetic changes in exhausted T cells, we performed an assay for transposase-accessible chromatin with sequencing (ATAC-seq) to assess chromatin accessibility.^44^ Differentially accessible peaks were annotated in the samples and transcription factor motif foot printing analysis was performed using TOBIAS and Hint.^45^^,^^46^ The transcription factor motifs that were significantly increased using both methods were compared between the *in vitro* activated and exhausted plus the *in vivo* TILs and *in vitro* activated, yielding a total of 25 that were shared in both the *in vitro* and *in vivo* exhausted CD8 T cells (Figure 5D). Interestingly, *Hoxa2* was previously shown to be enriched in exhausted T cells from the LCMV model.^47^ Many of other transcription factors shared between the *in vitro* exhausted and the TILs of mouse and human represent innovative areas for further study.

## Discussion

A greater knowledge of the dynamics of T cell exhaustion is crucial to enhance the efficiency of future immunotherapies. However, developing a deeper understanding of the molecular, morphological, and functional characteristics of exhausted T cells is hampered by the lack of a reliable method to produce these cells for study in tractable *ex vivo* models. Here, we present an *in vitro* model that allows direct comparison with TILs from both *in vivo* murine models and human studies. This model is a fast and reproducible system to study exhaustion in the laboratory by chronic antigenic stimulation in the absence of any other components of the tumor microenvironment. Other models of T cell exhaustion specific to viral infection^40^ and non-specific TCR stimulation mediated by antibodies^48^ have emerged as a useful tool to perform CRISPR based screenings, as alternatives to *in vivo* models. However, they have not characterized the temporal acquisition or diversity of the features of exhaustion that we have undertaken. The model presented here recapitulates the hallmarks of exhaustion, high expression of IRs, decreased proliferation, decreased cytokine production, and the alteration of metabolic and mitochondrial function,^1^^,^^6^^,^^19^^,^^20^ confirming that this is a reliable system to study exhaustion. Temporal analysis allowed the identification of the different phases in the development of exhaustion, and alluded to the sequential order of the alterations present in this population.

It is established that upon T cell activation, metabolic alterations result in increased mitochondrial mass and ROS^49^^,^^50^ and exhausted cells exhibited a further increase in both when compared with activated cells. Exhausted cells had a higher uptake of glucose and a decrease in the production of IFNγ, TNFα, and CD107a, highlighting a dysfunctional state. The importance of mitochondrial activity in exhausted cells has been shown previously.^51^^,^^52^ Here, we demonstrate the sequential nature of these events within the exhausted cells, with changes in the morphology of mitochondria and cells. These changes are linked to the functional and metabolic state of the cells. A similar relationship between metabolic stress and altered mitochondrial morphology has previously been demonstrated with hypoxic cells.^8^^,^^9^ We noticed that after the third stimulation it was possible to observe electron-dense structures that looked similar to a mitochondrial-lysosome-related organelle (MLRO) that had been described in hepatocytes dedifferentiation.^53^ However, more characterization is needed to confirm if these structures do exist in exhausted T cells and a potential role.

Our *in vivo* model allows the evaluation of different immune responses against the same tumor model expressing the OVA antigen, and permits the exploration of the immune populations involved in different responses. As in our model, a high infiltration of regulatory T cells (CD4^+^ FOXP3+) and increased CD4s have been shown to be present in non-responsive and regressing human tumors respectively,^54^^,^^55^^,^^56^^,^^57^ demonstrating the validity of our model. The comparison of *in vitro* and *in vivo* exhausted cells allows the identification of drivers of the exhaustion phenotype that are shared *in vitro* and *in vivo* and which are the genes that are specific to each condition. TILs from non-responsive and exhausted tumors expressed higher levels of key markers of exhaustion and increased mitochondrial mass when compared to the TILs from the regressing tumor response. These differences in mitochondria and exhaustion markers *in vivo* were similar to our observations *in vitro* when comparing exhausted and activated T cells. The high potential of the mitochondria has been associated with a dysfunctional state previously both *in vitro* and *in vivo.*^58^^,^^59^ However, other studies have associated the dysfunctional state with depolarized mitochondria^52^ in exhausted cells. This discrepancy could potentially be explained by differences in experimental techniques, our measurements were direct using TMRE while other studies have utilized Mitotracker stains. TILs from the regressing tumors had increased glucose uptake compared with TILs from the non-responders and exhausted tumors. In contrast, the exhausted cells *in vitro* had a higher glucose uptake compared with activated cells. This differential response could be due to the differences between the metabolic requirements of the TILs and the availability of glucose within tumor microenvironment.

As many of the differentially regulated genes in exhaustion can also be present in activated T cells all comparisons were made against recently activated T cells, this allows the identification of exhaustion specific drivers rather than those that play a role in T cell activation. One of the possible limitations of the *in vitro* model is the lack of any other components of the TME and the only driver for the induction of exhaustion being chronic TCR signaling. However, given the overlap from the RNAseq of 2501 upregulated genes there is a core set shared between the *in vitro* and *in vivo* models independent of the TME that allows to identify key genes or pathways from the unique up or down regulated genes in the relevant signatures (Tables S3 and S4). To further support the validity and translational potential of the model we identified 259 genes present in our core experimental signature that are also enriched within exhausted CD8 T cells isolated from a panel of human tumors.^32^ The genes found in the exhausted signature contained well established genes previously linked to exhaustion, including: *Pdcd1*, *Havcr2* and *Entpd1*. As well as those identified more recently through CRISPR screens including *Bhlhe40*^40^*,* and *Rbpj*.^41^The latter two examples are transcription factors that promote differentiation of exhausted T cells and there were in total 25 shared upregulated transcription factors between mouse and human. The pathways that are enriched in this shared exhaustion signature are related to cytokines, interleukins and TNF receptors that are vital for the development of the T cell exhaustion phenotype, and are part of the extrinsic drivers of T cell exhaustion.^60^ The Rho GTPases are pathways enriched in the experimental signature of exhaustion that have a critical role in T cell activation.^61^

Epigenetic changes are also critical in the development and maintenance of the exhausted state^6^^,^^34^^,^^42^^,^^43^ and we have identified 25 transcription factor motifs that have increased accessibility in both the *in vitro* model and *in vivo* TILs. This includes *Hoxa2*, which has previously been linked to virally exhausted T cells,^47^ with many others representing unexplored avenues to investigate their role in T cell exhaustion. In summary we present an *in vitro* model and key datasets that could be utilised in the development of targets for T cell reinvigoration in cancer immunotherapy.

### Limitations of the study

While this model offers a valuable tool for studying T cell exhaustion, it has limitations. Inevitably, it lacks the complex signaling cues provided by the tumor microenvironment, and some key metabolic challenges such as hypoxia and nutrient deprivation. While we did compare to two *in vivo* tumor models, this was at an early timepoint to avoid the tumor regression observed in the MC38 model. The consequence of this timepoint is T cell exhaustion is not fully established and thus it would be of interest in future studies to compare *in vitro* generated exhausted cells to T cells from more models *in vivo* at different timepoints. Our *in vivo* analysis also focused on the bulk CD8 T cell population rather than gating or enriching for exhausted cells for the analysis. It will be of interest to follow up the effect of PD1/PD-L1 blockade or other proposed therapies in this model. One other aspect of T cell exhaustion that was not directly measured in this study is the cytotoxic capacity, either *in vitro* or *in vivo*. The strong TCR signal of the OT-I T cells used here induces rapid exhaustion.^62^ This makes the model well-suited for examining responses to cognate antigens in cancer, but unsuitable for studying the nuanced process of T cell exhaustion mediated by low-affinity TCR binding. To isolate true exhaustion markers, comparisons in this study were made against activated T cells, ensuring that identified markers are specific to exhaustion and not simply a consequence of T cell activation.

## Resource availability

### Lead contact

Requests for further information and resources should be directed to Anneliese O. Speak (as3234@cam.ac.uk).

### Materials availability

This study did not generate new unique reagents. Plasmids and/or cell lines generated in this study are available upon reasonable request to the lead author.

### Data and code availability

The data for this study have been deposited in the European Nucleotide Archive (ENA) at EMBL-EBI under accession number PRJEB77527 (https://www.ebi.ac.uk/ena/browser/view/PRJEB77527) for RNA sequencing and PRJEB41099 (https://www.ebi.ac.uk/ena/browser/view/PRJEB41099) for ATAC sequencing. This paper does not report original code. Any additional information required to reanalyze the data reported in this paper is available from the lead contact upon request.

## Acknowledgments

We thank Dr Simon Clare and Katherine Harcourt for technical support. We acknowledge the services provided by the NIHR Cambridge BRC Cell Phenotyping Hub, as well as the Cytometry Core Facility, DNA pipelines and Informatics at the Wellcome Sanger institute (supported by grant WT206194). We are grateful for the facilities and support provided by the Wellcome Sanger Institute Research Support facility and University of Cambridge University Biological Services Unit. We thank Dr Holly Robertson, Dr Victoria Harle, and Dr Vincent Zecchini for their careful reading and helpful comments on the manuscript and Dr Juan Pablo Narvaéz Goméz for his help with code and independent measurements of mitochondria. A.J.M.-R. was supported by a grant from Open Targets (grant OTAR2049), B.F. and A.O.S. were supported by a package from Wellcome Sanger Institute and 10.13039/100010269Wellcome Trust (grant WT206194). D.A.G. was supported by the 10.13039/100010269Wellcome Trust, S.H. was supported by Open Targets (grant OTAR3000), and D.J.A. was supported by the 10.13039/100010269Wellcome Trust (grant WT206194).

## Author contributions

Conceptualization, A.J.M-R. and A.O.S.; Methodology, A.J.M-R., B.F., D.A.G. and A.O.S.; Formal analysis, A.J.M-R., S.H. and A.O.S.; Investigation, A.J.M-R., B.F., D.A.G. and A.O.S.; Resources, S.H., D.A.G., D.J.A. and A.O.S.; Supervision, D.J.A. and A.O.S.; Funding acquisition, D.J.A. and A.O.S.; Writing – original draft, A.J.M-R. and A.O.S.; Writing – review & editing, all authors.

## Declaration of interests

No authors have relevant conflicts of interest to declare.

## STAR★Methods

### Key resources table

**Table:** 

REAGENT or RESOURCE	SOURCE	IDENTIFIER
**Antibodies**		

Anti-mouse CD366 (Tim-3) PE/Cyanine7	Biolegend	Cat# 134010; RRID:AB_2632734
Anti-mouse CD223 (LAG-3) PerCP/Cyanine5.5	Biolegend	Cat# 125212; RRID:AB_2561516
Anti-mouse CD45 Alexa Fluor® 700	Biolegend	Cat# 103128; RRID:AB_493715
Anti-mouse CD39 Vio® Bright B515	Miltenyi Biotec	Cat# 130-114-361; RRID:AB_2784174
Anti-Mouse CD244.2 BV786	BD Biosciences	Cat# 740860; RRID:AB_2740513
Anti-Mouse CD279 (PD-1) BV421	BD Biosciences	Cat #569780; RRID:AB_3685317
Anti-Mouse CD152 (CTLA-4) APC	Miltenyi Biotec	Cat #130-116-455; RRID:AB_2727554
Anti-Mouse Ly-108 BV711	BD Biosciences	Cat # 740823; RRID:AB_2740481
Anti-Mouse CD69 BV650	BD Biosciences	Cat# 569688; RRID:AB_3685232
Anti-Mouse CD8a APC-H7	BD Biosciences	Cat# 560182; RRID:AB_1645237
Anti-Mouse IFN-γ BV421	BD Biosciences	Cat# 563376; RRID:AB_2738165
Anti-mouse CD107a (LAMP-1) PE	Miltenyi Biotec	Cat# 130-111-504; RRID:AB_2654463
Anti-mouse TNF-α PE/Cyanine7	Biolegend	Cat# 506324; RRID:AB_2256076
Anti-human/mouse Granzyme B PerCP/Cyanine5.5	Biolegend	Cat# 372212; RRID:AB_2728379
Anti-mouse CD357 (GITR) BV421	BD Biosciences	Cat# 563391; RRID: AB_2738177
Anti-Mouse CD279 (PD-1) PE	Miltenyi Biotec	Cat# 130-111-800; RRID:AB_2656934
Anti-Mouse CD152 (CTLA-4) PE-CF594	BD Biosciences	Cat#564332; RRID:AB_2732917
Anti-Mouse CD69 PE-CF594	BD Biosciences	Cat#562455; RRID:AB_11154217
Anti-Mouse CD279 BUV737	BD Biosciences	Cat#749422; RRID:AB_2873791
Anti-Mouse CD69 FITC	Miltenyi Biotec	Cat#130-115-459; RRID:AB_2727051
Anti-Mouse T-bet BV785	Biolegend	Cat#644835; RRID:AB_2721566
Anti-Mouse TOX PE	Miltenyi Biotec	Cat#130-120-716; RRID:AB_2801780
Anti-Mouse TCR β BUV496	BD Biosciences	Cat# 749915; RRID:AB_2874154
Anti-Mouse CD4 Viogreen	Miltenyi Biotec	Cat#130-118-693; RRID:AB_2801780
Anti-Mouse CD8 APC	Miltenyi Biotec	Cat#130-117-664; RRID:AB_2728016
Anti-Mouse CD45 Alexa Fluor 700	Biolegend	Cat#103128; RRID:AB_493715
Anti-mouse CD39 Vio® Bright B515	Miltenyi Biotec	Cat#130-114-252; RRID:AB_2726515
Anti-Mouse CD16/CD32 (Mouse BD Fc Block)	BD Biosciences	Cat#553142; RRID:AB_394656
Anti-mouse CD107a (LAMP-1) PerCP/Cyanine5.5	Biolegend	Cat#121625; RRID: AB_2572055

**Chemicals, peptides, and recombinant proteins**		

Dulbecco′s Phosphate Buffered Saline	Sigma-Aldrich	Cat# D8537
Fetal Bovine Serum	Thermofisher	Cat# A5670701
RPMI 1640 Medium, no glucose	Thermofisher	Cat# 11879020
Penicillin-Streptomycin-Glutamine (100X)	Thermofisher	Cat# 10378016
Recombinant Human IL-2 Protein	PeproTech	Cat# 200-02
MEM Non-Essential Amino Acids Solution (100X)	Thermofisher	Cat# 11140050
Sodium Pyruvate (100 mM)	Thermofisher	Cat# 11360039
2-Mercaptoethanol (50 mM)	Thermofisher	Cat# 31350010
Fixable Viability Dye eFluor 780	Thermofisher	Cat# 65-0865-14
DAPI	Merck Life Science	Cat# MBD0015-5ML
MitoTracker deep red	Thermofisher	Cat# M22426
MitoStatus TMRE - 25 mg	BD Biosciences	Cat# 564696; RRID AB_2869606
BD GolgiStop	BD Biosciences	Cat# 554724
Mouse IL-15 Recombinant Protein	Thermofisher	Cat# 210-15-100UG
Fixable Viability Dye eFluor 455UV	Fisher scientific	Cat# 15510617
OVA peptide 257-264	Invivogen	Cat# vac-sin
HEPES (1M)	Thermofisher	Cat# 15630080
DMSO, Anhydrous	Thermofisher	Cat# D12345
DMEM, high glucose, no glutamine, no methionine, no cystine	Thermofisher	Cat# 21013024
Tween 20 (10%)	Merck Life Science	Cat# 11332465001
NP40 (10%)	Merck Life Science	Cat# 11332473001
Digitonin	Promega	Cat# G9441

**Critical commercial assays**		

LS Columns	Miltenyi Biotec	Cat# 130-042-401
CD8a+ T cell Isolation Kit, mouse	Miltenyi Biotec	Cat# 130-104-075
Click-iT Plus EdU Alexa Fluor 647 Flow Cytometry Assay Kit	Thermofisher	Cat# C10635
CellROX Deep Red Reagent, for oxidative stress detection	Thermofisher	Cat# C10422
2-NBDG (2-(N -(7-Nitrobenz-2-oxa-1,3-diazol-4-yl)Amino)-2-Deoxyglucose)	Thermofisher	Cat# N13195
OSMIO	N/A	N/A
Cytofix/Cytoperm Fixation/Permeablization	BD Biosciences	Cat# 554714
Foxp3/Transcription Factor Staining Buffer Set	Thermofisher	Cat# 00-5523-00
BD Horizon Brilliant Stain Buffer	BD Biosciences	Cat# 563794
Tumor Dissociation Kit, mouse	Miltenyi Biotec	Cat# 130-096-730
CD4/CD8 (TIL) MicroBeads, mouse	Miltenyi Biotec	Cat# 130-116-480
Seahorse Cell Mito Stress Test Kit	Agilent technologies	Cat# 102601-100
TRIzol	Invitrogen	Cat# 15596026
Direct-zol RNA Microprep Kits	Zymo research	Cat# R2060
Illumina Tagment DNA Enzyme and Buffer	Illumina	Cat# 20034197
Nextera XT DNA Library Preparation Kit	Illumina	Cat# FC-131-1096
Nextera Index kit	Illumina	Cat# FC-121-1011
Monarch® Spin PCR & DNA Cleanup Kit	New England Biolabs	Cat# T1130
Agilent High Sensitivity DNA Kit	Agilent	Cat# 5067-4626

**Deposited data**		

RNA sequencing data	European Nucleotide Archive	Accession number: ERP161925
ATAC sequencing data	European Nucleotide Archive	Accession number: ERP124831

**Experimental models: Cell lines**		

B16F10	ATCC	Cat#: CRL-6475; RRID:CVCL_0159
MC38	gift from L. Borsig	RRID:CVCL_B288

**Experimental models: Organisms/strains**		

OT-1 (C57BL/6-Tg(TcraTcrb)1100Mjb/J)	Jackson Laboratories	RRID:IMSR_JAX:003831
FIR (C57BL/6-*Foxp3*^*tm1Flv*^*/*J)	Jackson Laboratories	RRID:IMSR_JAX:008374

**Recombinant DNA**		

PCI-neo-cOVA	Gift from M. Castro	RRID:Addgene_25097
PB-CMV-MCS-EF1a-Puro PiggyBac vector	System Biosciences	PB501b-1
piggybac transposon	Gift from A. Bradley	N/A

**Software and algorithms**		

R version 4.3.0	The R Foundation	https://www.r-project.org/
FlowJo 10	FlowJo LLC	RRID: SCR_008520
Graphpad Prism	Graphpad Software	RRID: SCR_002798

### Experimental model and study participant details

#### Mice

Wildtype mice were obtained from genetically altered C57BL/6NTac lines from the Wellcome Sanger Institute Mouse Genetics Project or Taconic. OT-I (C57BL/6-Tg(TcraTcrb)1100Mjb/J, RRID:IMSR_JAX:003831) and FIR (C57BL/6-*Foxp3*^*tm1Flv*^*/*J, RRID:IMSR_JAX:008374) mice were obtained from The Jackson Laboratories. Animals were housed in Sanger Institute Research Support Facility and University of Cambridge Biological Services Unit under Specific Opportunist Pathogen Free conditions in Individually Ventilated Cages at a density of 1–6 animals per cage. Mice had *ad libitum* access to irradiated or autoclaved diet and autoclaved water with aspen bedding and environmental enrichment provided. The care and use of all mice in this study were in accordance with the Home Office guidelines of the UK and procedures were performed under a UK Home Office Project License (P2E57E159) which was reviewed and approved by the Sanger Institute’s and University of Cambridge Animal Welfare and Ethical Review Body. For *in vitro* and *in vivo* experiments mice were between 7 and 20 weeks old at experimental initiation and mice of both sexes were used. For the tumor growth studies cohorts of sizes between 20 and 70 animals size dependent on sex and age matching and expected dosing groups to achieve sufficient replicates of each result. Where possible, age and sex matched mice in the cohorts were littermates and both sexes were used to reduce sex bias and the metadata is in Table S2. Sex of the mice was not accounted for in the statistical analysis.

#### Cell lines

MC38 cells (a gift from L. Borsig) and B16-F10 cells (from ATCC, CRL-6475) were tested for mycoplasma (IDEXX labs) and mouse pathogens (Charles River Laboratories). The B16-F10 cell line was authenticated by whole genome sequencing and the MC38 cell line was not authenticated.

### Method details

#### Reagents

Unless otherwise stated tissue culture reagents and other biochemicals were from Sigma-Aldrich and ThermoFisher.

#### *In vitro* exhaustion protocol

CD8 T cells were isolated from spleens of OT-I mice using the CD8 mouse isolation kit (Miltenyi Biotec) according to the instructions of the manufacturer. Cells were counted and seeded at a density of 1x10^6^ per mL in complete media: RPMI 1640 supplemented with 10% heat inactivated fetal bovine serum (FBS), 50 μM 2 mercaptoethanol, 1% non-essential amino acids, 1 mM sodum pyruvate, 10 mM HEPES, 2mM L glutamine, 100 U/mL penicillin and 100 μg/mL streptomycin (CM). The OVA peptide SIINFEKL (AnaSpec) was added at 3 μg/mL with 100 U/mL human IL-2 (Peprotech). Every 48 h the cells were centrifuged and the pellet was resuspended between 1 and 2 x 10^6^ per mL in fresh complete media containing the OVA peptide 3 μg/mL and IL-2 (100 U/mL) after the second stimulation IL-15 (10 ng/mL, Peprotech) was added The number of stimulations is indicated in the figure/text. Comparisons were made with CD8 T cells activated for 24h with OVA (3 μg/mL) in CM with 100U/mL of IL-2 and expanded for 72h if indicated.

#### Proliferation with 5-Ethynyl-2-deoxyuridine (EdU)

Cells were incubated with EdU (10 μM) for one hour in CM, then stained for viability with the live/dead fixable red stain according to manufactures instructions. Cells were fixed with 4% PFA for 15 min at room temperature. Then washed with permeabilization buffer from the Foxp3/Transcription Factor Staining Buffer Set (eBioscience) and incubated in 10 mM sodium ascorbate, 2 mM copper sulfate and 1 μM Alexa 647 azide dye for 30 min at room temperature. After washing, cells were labeled with 2 μg/mL Hoechst 33452 for 15 min. Cells were then analyzed by flow cytometry on a Fortessa SORP instrument (BD Biosciences).

#### Preparation of tissue samples for sorting and FACS

Tumors were collected and processed using the tumor dissociation kit (Miltenyi Biotech) and GentleMACS C tubes using a GentleMACS dissociator according to the supplied protocol for soft//medium tumors. T cells from tumor single cell suspensions were enriched with the CD8/CD4 (TIL) MicroBeads kit according to the manufacturer’s instructions (Miltenyi Biotech). Lymph nodes and spleens were crushed through a 70 μm filter (MACS Smart Strainer) and washed with PBS. Cells from spleens were treated with red blood cell lysis, centrifuged and washed with PBS.

#### Flow sorting

After tissue processing to single cell suspension, cells were stained with CD8a and eBioscience Fixable Viability Dye eFluor 780. Cell sorting was performed on a BD FACS Aria with a 100 μm nozzle.

#### Flow cytometry

For surface membrane markers cells were resuspended in titrated amounts of antibodies diluted in FACS buffer (D-PBS without calcium and magnesium, 0.5% FBS, 2 mM EDTA and 0.09% sodium azide) and incubated in the fridge for 30 min. Viability was determined using a compatible dye (eBioscience Fixable Viability dye eFluor 780, eFluor 506, eFluor 455UV or DAPI). Where required for intracellular staining cells were fixed with an appropriate fixative either Foxp3/Transcription Factor Staining Buffer Set (eBioscience) or Cytofix/Cytoperm (BD) and intracellular staining performed in the appropriate permeabilisation buffer (either for 45 min or overnight in the fridge). Samples were analyzed on a MACSQuant VYB, BD Fortessa, Cytex Aurora and Sony ID7000. Compensation samples were prepared with Ultracomp eBeads (eBioscience), Arc reactive beads (Invitrogen), anti-REA Comp beads (Miltenyi Biotec) and unstained cells and determined on the instrument using inbuilt software.

#### Degranulation assays to evaluate cytokine production

Cells were seeded in a 96 well plate with CM media containing the protein transport inhibitor monensin (BD GolgiStop, 1 in 1000) and CD107a antibody (0.75 μg/mL), with or without OVA (2 μg/mL) for four hours at 37°C in 5% CO_2_. Cells were stained with eBioscience Fixable Viability dye eFluor 780 and fixed with Cytofix/Cytoperm solution for 20 min at 4°C. After washing with perm/wash solution (BD, USA) cells were stained for 30 min at 4°C. Samples were acquired on a flow cytometer (MACSQuant VYB, BD Fortessa or Cytek Aurora).

#### Metabolic stress test

Exhausted and activated T cells were seeded on a poly-D-lysine-coated 96-well plate (200,000 cells per well). T cell fitness standard kit test (Agilent, Santa Clara, CA) was performed on a Seahorse XFe96 Analyzer. Metabolic rate was normalized to cell count determined on a Moxi (Orflo).

#### Mitochondria and metabolism profiling through FACS

Cells were seeded in 96 well plates and stained using: reactive oxygen species (CellROX Deep Red Reagent, 5 μM), mitotracker (MitoTracker Deep Red FM, 2 μM) and TMRE (Tetramethylrhodamine ethyl ester, 1 μM) in complete media. To measure glucose uptake cells were seeded in growth media without glucose with the addition of 20 μM 2-NBDG (2-(*N*-(7-Nitrobenz-2-oxa-1,3-diazol-4-yl)Amino)-2-Deoxyglucose). Cells were incubated for 30 min at 37°C in 5% CO_2_ then washed and stained with DAPI (200 ng/mL) for viability and acquired on a flow cytometer (MACSQuant VYB, BD Fortessa or Cytek Aurora).

#### Confocal microscopy

Cells were seeded in bottom glass dishes and stained with nucblue (2 drops per 1 mL of NucBlue Live ReadyProbes Reagent (Hoechst 33342) ThermoFisher) and mitotracker (MitoTracker Deep Red FM, 2 μM ThermoFisher). Cells were acquired in a confocal microscope Leica SP8 and processed with the Leica Application Suite.

#### Electron microscopy

T cells were pelleted and fixed (using a solution of 2.5% glutaraldehyde and 2% paraformaldehyde in 0.1 M sodium cacodylate buffer) at isolation (Naive) and every 48 h after stimulation. Cells were post-fixed with 1% osmium tetroxide and mordanted with 1% tannic acid, followed by dehydration using an ethanol series (contrasting with uranyl acetate at the 30% stage) and embedding with an Epoxy Resin Kit (Sigma-Aldrich). Ultrathin sections were cut on a Leica UC6 ultramicrotome contrasted with lead citrate and images were acquired on a FEI Spirit Biotwin, using a Teitz FX416CCD Tem Cam.

#### *In vivo* experiments

OVA expressing tumor cell lines were generated by inserting the cOVA sequence from PCI-neo-cOVA (a gift from M. Castro, RRID:Addgene_25097), into PB-CMV-MCS-EF1a-Puro PiggyBac vector (PB501b-1, System Biosciences) to generate PB-CMV-cOVA-EF1a-Puro plasmid which was delivered together with piggybac transposon (a gift from A. Bradley) to MC38 or B16-F10 cells. After selection with puromycin (2 μg/mL, Invivogen) expression of OVA was determined by western blotting (Abcam) and presentation of SIINFEKL peptide in the context of MHC Class I on the surface of tumors cells was determined by flow cytometry (H-2K^b^/SIINFEKL, REA1002, Miltenyi Biotec) and the cell lines were maintained as a pool. For early phenotypic assays FIR mice (split equally between males and females) were subcutaneously injected with 3 x 10^5^ B16-F10-OVA or 2 × 10^6^ MC38-OVA to the flank and tumors, spleens or draining lymph nodes harvested after 9 days. Wild type or FIR mice received between 1.5 and 2.0 x 10^6^ MC38-OVA or MC38 parental cells subcutaneously (exact cell numbers for each cohort are detailed in the Table S2). Tumor measurements were collected at regular intervals by one individual using a caliper and tumor area calculated (length x width (cm)). Tissue collection on some of the mice for flow cytometry analysis was performed on days 15, 16 and 17 (Table S2) and the cohort of mice was classified on the day of tissue collection or day 17 whichever was sooner. For adoptive transfer experiments WT mice were injected subcutaneously with MC38-OVA 2 x 10^6^ cells. On day 16 post tumor cell administration OT-I cells that had been activated *in vitro* with OVA (2 μg/mL) for 24 h prior were injected via tail vein injection (5 x 10^6^ cells/mouse) and tissues harvested 21 days after T cell transfer (day 37 post tumor cell injection).

#### RNAseq

Cells were processed in Trizol (Life Technologies) and total RNA was isolated using the RNA Clean and Concentrator (Zymo Research). Libraries were prepared using the Illumina stranded total RNA prep with Ribo-Zero plus kit according to the manufacturer’s instructions and sequenced on an Illumina HiSeq 4000 with 75 bp paired end reads aiming for >30 million reads per sample.

#### ATACseq

10,000 sorted cells were treated as previously described^44^ using the Illumina Tagment DNA TDE1 Enzyme and Buffer Kits according to the manufacturer’s instructions. DNA was cleaned using the Monarch kit (NEB) and quantified using a bioanalyzer (Agilent). Nextera adapters were added using Nextera XT DNA library preparation kit with 9 PCR cycles performed. Samples were sequenced on an Illumina HiSeq 4000 with 50 bp paired end reads.

### Quantification and statistical analysis

#### Flow cytometry

Data was analyzed with FlowJo software (v10.7.1, BD) using singlets and viable cells for the *in vitro* experiments and for the *in vivo* experiments using the gating strategy outlined in Figure S9.

#### Imaging

Analysis of EM images to determine, area and circularity of cells and mitochondria was done using the open source project for image analysis ImageJ, also known as Fiji following the methods described by Lam et al.^63^ Fourteen random samples were blinded measured and counted by three independent researchers to confirm results. The formula for circularity is 4π(area/perimeterˆ2). A value of 1.0 indicates a perfect circle.

#### Tumor growth classification

Tumors were classified adopting the scripts from the INSPECTumours package^35^ as outlined below. A minimum of 3 measurements from day 10 was required for the classification. Tumor area (cm2) was converted to tumor area (mm2, multiplication by 100) and if tumors were undetectable the value was set to 1 to enable log10 transformation. Mice were grouped into ‘control’ or ‘treated’ required for the prediction model by deriving the area under the curve for the tumor growth area (DescTools AUC function) and assigning the mice in the top quartile as ‘control’. A two-stage non-linear model covering 75% of the assigned ‘control’ mice up to day 17 was used to generate the predicted tumor growth (model_control, run_nl_model and predict_nlm_single functions) and to classify each timepoint as a responder or non-responder (classify_data_point and get_responder functions). The classification on the final day of measurement (responder or non-responder) as well as the total of responder or non-responder classifications from day 10 were used to classify the tumor into a responder or non-responder. The growth rate of each tumor from day 10 was calculated (calc_gr function) and a *p* value determined using a Student’s t test according to the package. For the final grouping if the tumor was classified as a non-responder it was called non-responsive, in addition if the final datapoint was classified as non-responder it was also called non-responsive. For those tumors that were responder they were subclassified as regressing if the growth rate was negative and *p* value less than 0.05, otherwise they were called exhausted.

#### RNAseq

Gzipped FastQ files were aligned to GCRm38 using star 2.7.9a,^64^ called via rsem 1.3.1^65^ (paired end mode, all other settings as default) to quantify transcript level counts. Differential expression was assessed using the R package DESeq2^66^ in R-4.1.0^67^ genes assigned an FDR<= 0.01 and displaying a log fold change of at least 2 were defined as significant. Reactome pathway enrichment was performed adapting the GSEA^37^ method by downloading the mouse Reactome annotation (MSigDB 2024.1, m2.cp.reactome.v2024.1.Mm.symbols.gmt). Genes were assigned Reactome annotations and the *p* value calculated using a hypergeometric distribution with the phyper function in R according to the original GSEA method. The total gene number in mouse was set to 42739 according to the MSigDB. *p* values were adjusted via the Benjamini & Hochberg method with FDR of 0.01 and ranking according to the fold enrichment used to filter gene lists of interest. Mouse to human mapping was performed using the annotations from the Alliance of Genome Resources obtained from the Mouse Genome Informatics website (https://www.informatics.jax.org/downloads/reports/index.html#homology, HOM_MouseHumanSequence.rpt) with transcription factor annotations obtained from FANTOM5 (https://fantom.gsc.riken.jp/5/sstar/Browse_Transcription_Factors_mm9) and merging EntrezGene via MGI id using a batch query on the Mouse Genome Informatics website.

#### ATACseq

FastQ files were aligned to GRCm38 using bwa 0.7.17 using the following flags mem -t 16 -p -Y -K 100000000. A PhiX alignment was also performed for filtering purposes and duplicate reads were marked using biobambam2 (2.0.79) and samtools. Peaks were called by analysing mapped bam files with macs2 callpeak MACS 2.2.6.1,^68^ with bigwig support enabled (i.e., with the - - bdg flag), a q value of 0.05 and –scale-to set to small. Footprint calling was performed using HINT (1.0.2) from the rgt package, more specifically “rgt-hint footprinting”, followed by “rgt-motifanalysis matching” and finally “rgt-hint differential”. Footprint analysis was performed using TOBIAS 0.17.0 via “TOBIAS ATACorrect”, followed by “TOBIAS FootprintScores” and finally “TOBIAS BINDetect”.

### Statistical analysis

Statistical comparisons were performed using Prism 10.0 (GraphPad) with the tests outlined in the figure legends and significant *p* values added to the text except Figures 3F–3J where all the *p* values are in the Table S1. *In vivo* experiments data were analyzed with Phenstat in R using the time as a fixed-effects model to allow pooling across multiple experiments with the PhenStat package^69^, p values were adjusted for multiple testing in R with the Benjamini & Hochberg method using the p.adjust function. Tumor growth curves were analyzed with the INSPECTumours package^35^ as described above.

## Supplemental information

Document S1. Figures S1S9

Table S1. Excel file containing results of the ANOVA tests with multiple comparisons related to Figure 3 F- Mitochondria count, G- Area of cells, H-Circularity of cells, I-area of mitochondria, J-Circularity of mitochondria.

Table S2. Excel file containing metadata and tumor measurements of *in vivo* experiments to establish response model relating to Figure 4A Cage indicates which mice were co-housed, Familial_Group indicates which mice had the same parents and where there is a letter it was consecutive litters. Dosing_date is when the cells were injected and these mice were all given the same batch of cells. Classification_day is the timepoint of tumor measurements for the INSPECT classification being applied, this was typically when a subset of mice were collected for analysis. Tumour_classification is the INSPECT classification for the tumor growth.

Table S3. Excel file containing signature gene annotation and Reactome pathway enrichment relating to Figure 5B

Table S4. Excel file containing list of transcription factors found in each signature relating to Figure 5D
