# Supplementary material for: Simulating CD8 T cell exhaustion: A comprehensive approach
Source: iScience. 2025 Jun 12;28(7):112897. doi: 10.1016/j.isci.2025.112897 (PMC12268933; doi:10.1016/j.isci.2025.112897)
Supplement: Document S1. Figures S1S9 [file mmc1.pdf]

iScience, Volume *28*

Supplemental information

Simulating CD8 T cell exhaustion: A comprehensive approach

Andrea J. Manrique-Rincón, Ben Foster, Stuart Horswell, David A. Goulding, David J. Adams, and Anneliese O. Speak

Figure S1

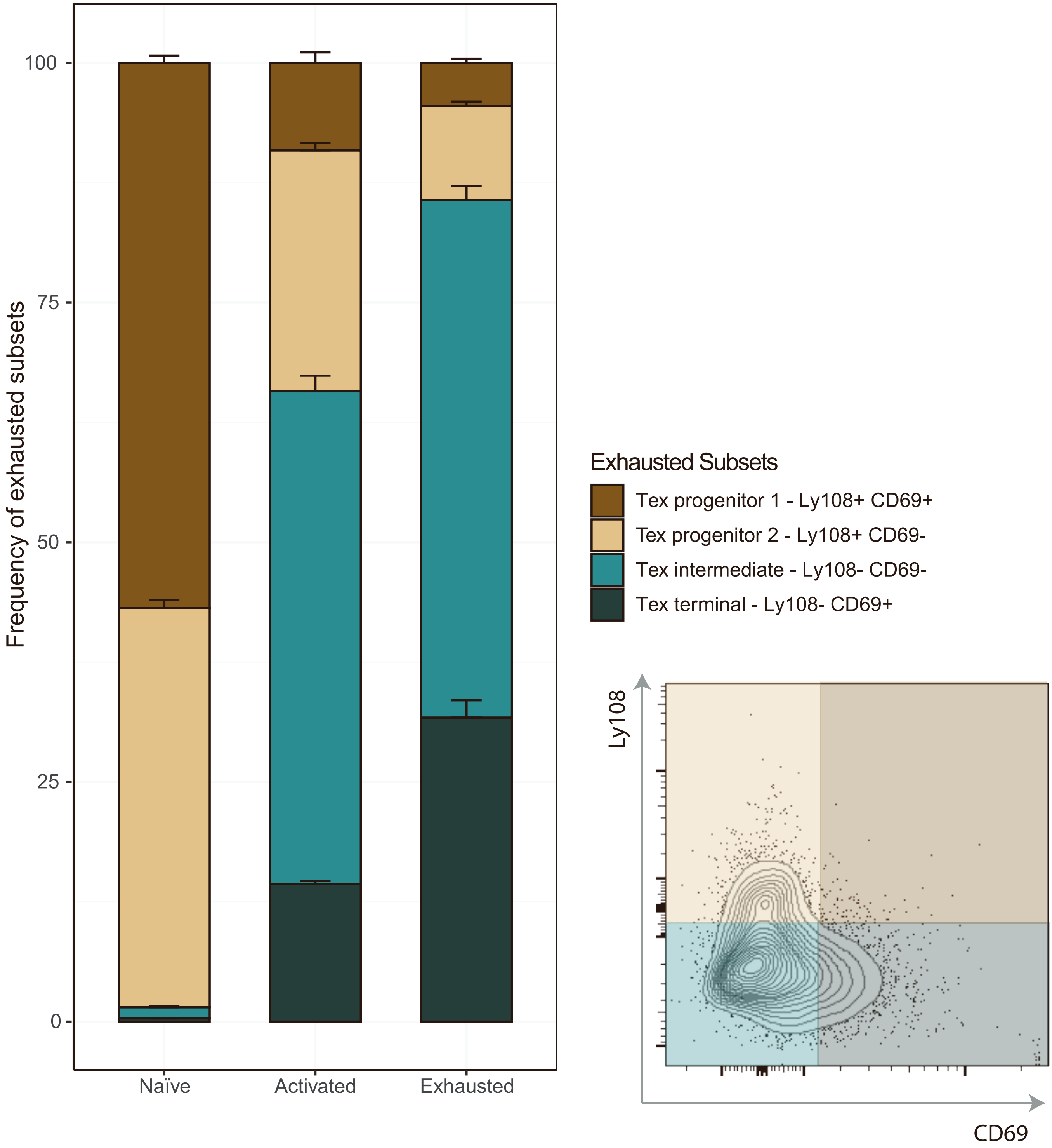

Frequency of exhausted subsets
100
75
50
25
0
Naïve
Activated
Exhausted
Exhausted Subsets
Tex progenitor 1 - Ly108+ CD69+
Tex progenitor 2 - Ly108+ CD69-
Tex intermediate - Ly108- CD69-
Tex terminal - Ly108- CD69+
Ly108
CD69

Figure S1. Exhausted subsets found *in vitro*, related to Figure 1.

Representative exhausted subsets as defined by CD69 and Ly108 expression in naïve, activated and exhausted T cells samples *in* vitro with representative gate. Mean SE, n=6.

Figure S2

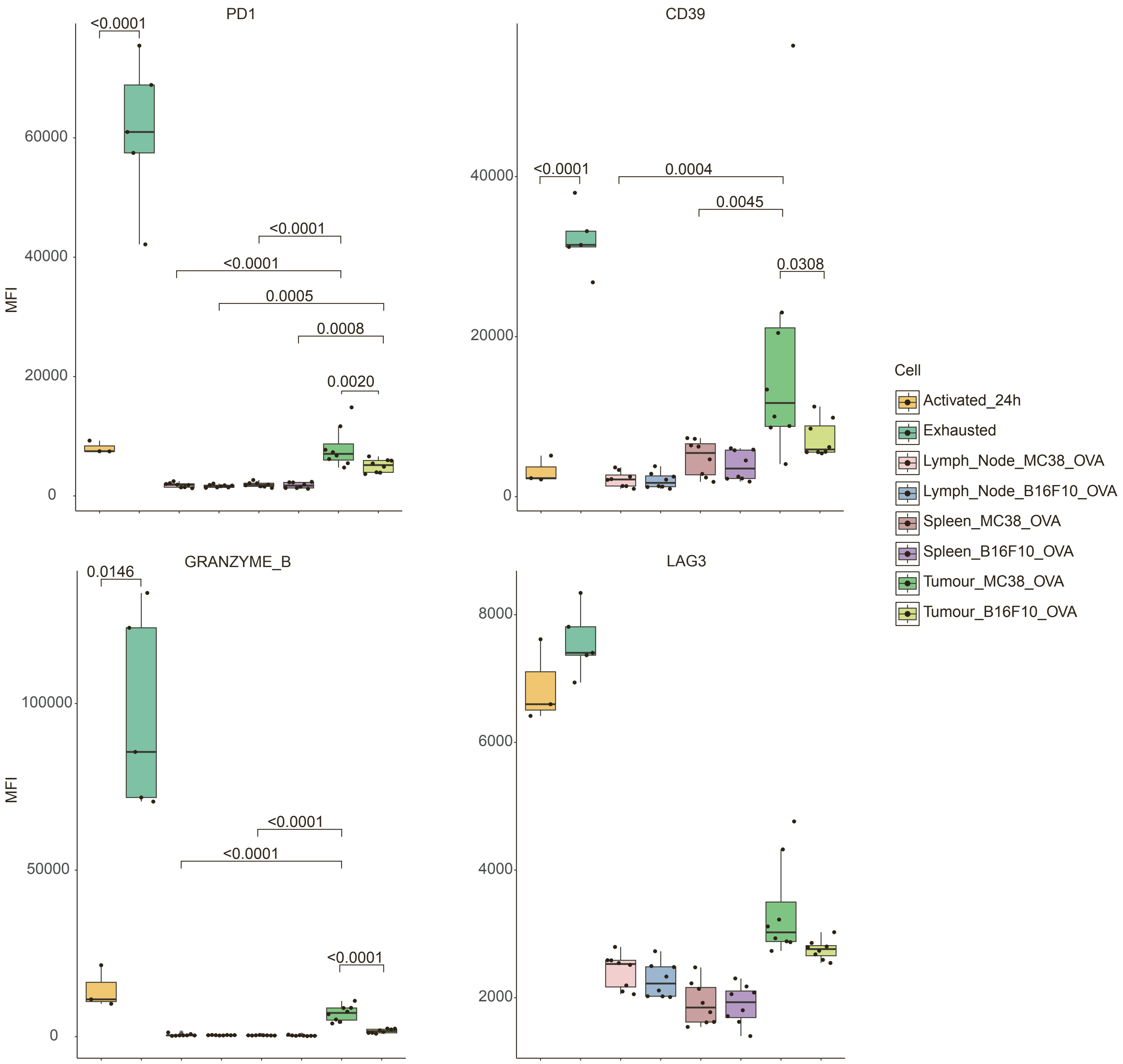

PD1
CD39
GRANZYME_B
LAG3
MFI
MFI
<0.0001
<0.0001
<0.0001
0.0005
0.0008
0.0020
0.0004
0.0045
0.0308
0.0146
<0.0001
<0.0001
<0.0001
60000
40000
20000
0
100000
50000
8000
6000
4000
2000
Cell
Activated_24h
Exhausted
Lymph_Node_MC38_OVA
Lymph_Node_B16F10_OVA
Spleen_MC38_OVA
Spleen_B16F10_OVA
Tumour_MC38_OVA
Tumour_B16F10_OVA

Figure S2. *In vivo* and *in vitro* exhaustion profile, related to Figure 1.

MFI of CD39, LAG3, PD1 and Granzyme B in activated, exhausted in vitro, lymph node, spleens and tumours of mice bearing B16F10-OVA and MC38-OVA tumours. Statistical analysis performed by a two-way ANOVA with a Sidak’s multiple-comparisons test.

Figure S3

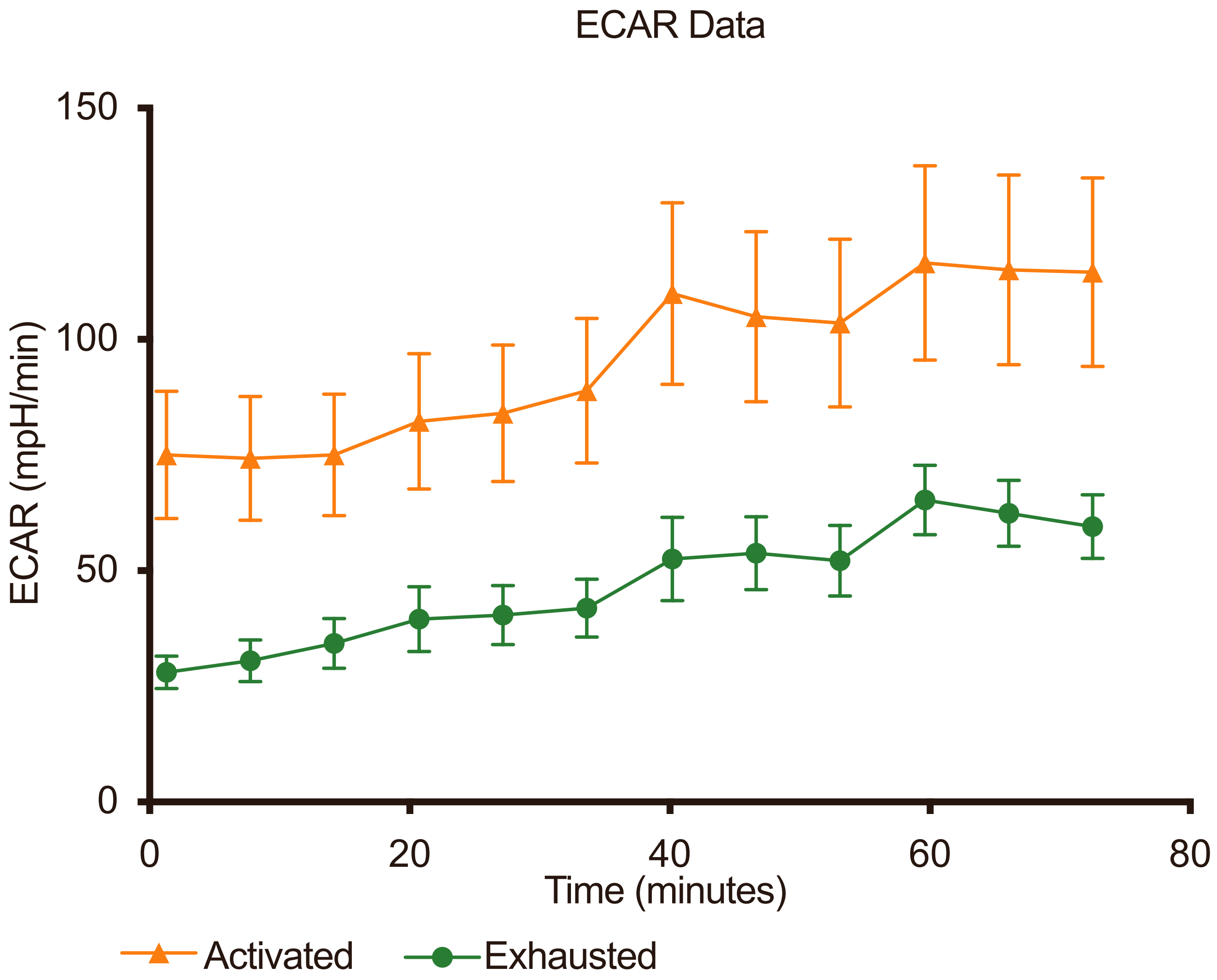

ECAR Data
150
100
50
0
ECAR (mpH/min)
0
20
40
60
80
Time (minutes)
Activated
Exhausted

Figure S3. ECAR profile, related to Figure 2.

Representative ECAR trace of exhausted and activated cells, Mean and SEM error, n=4.

Figure S4

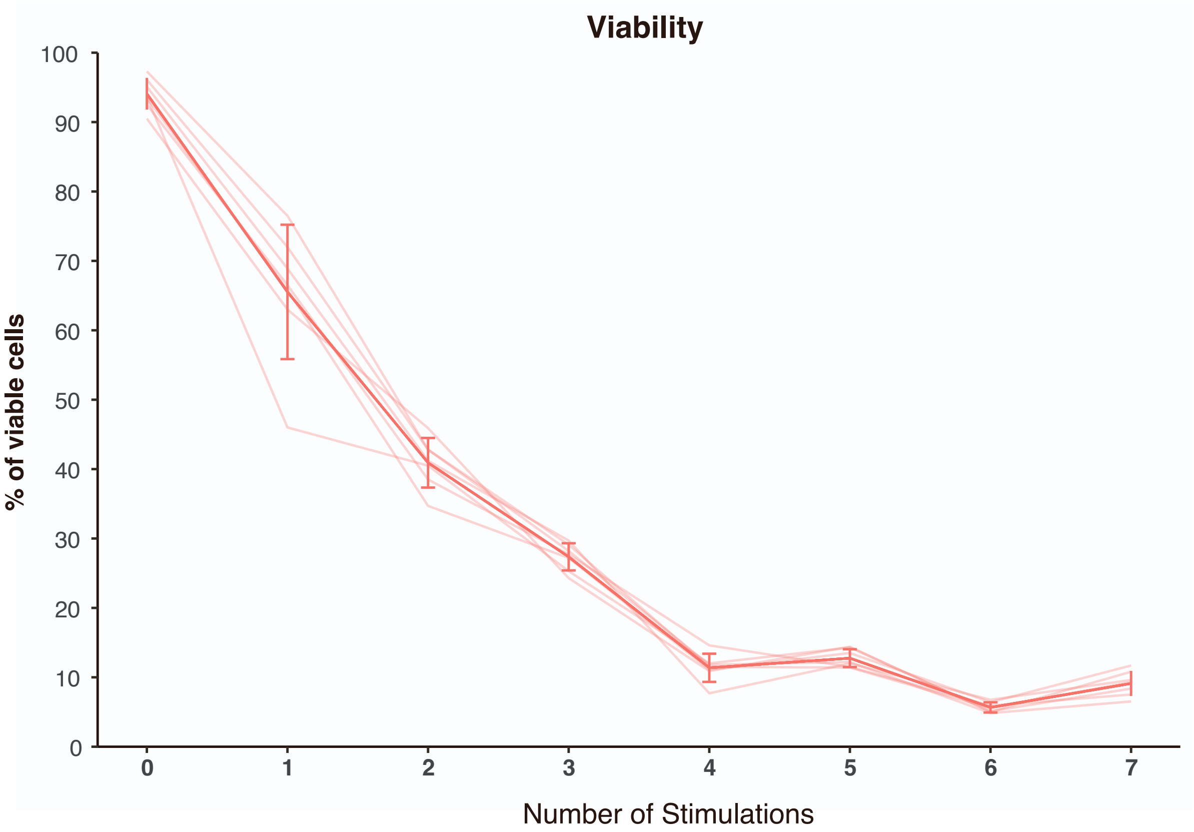

Viability
% of viable cells
100
90
80
70
60
50
40
30
20
10
0
0
1
2
3
4
5
6
7
Number of Stimulations

Figure S4. Time course of viability during *in vitro* exhaustion protocol, related to Figure 3.

Viability after each stimulation measured with fixable viability Dye eFluor 780.

Figure S5

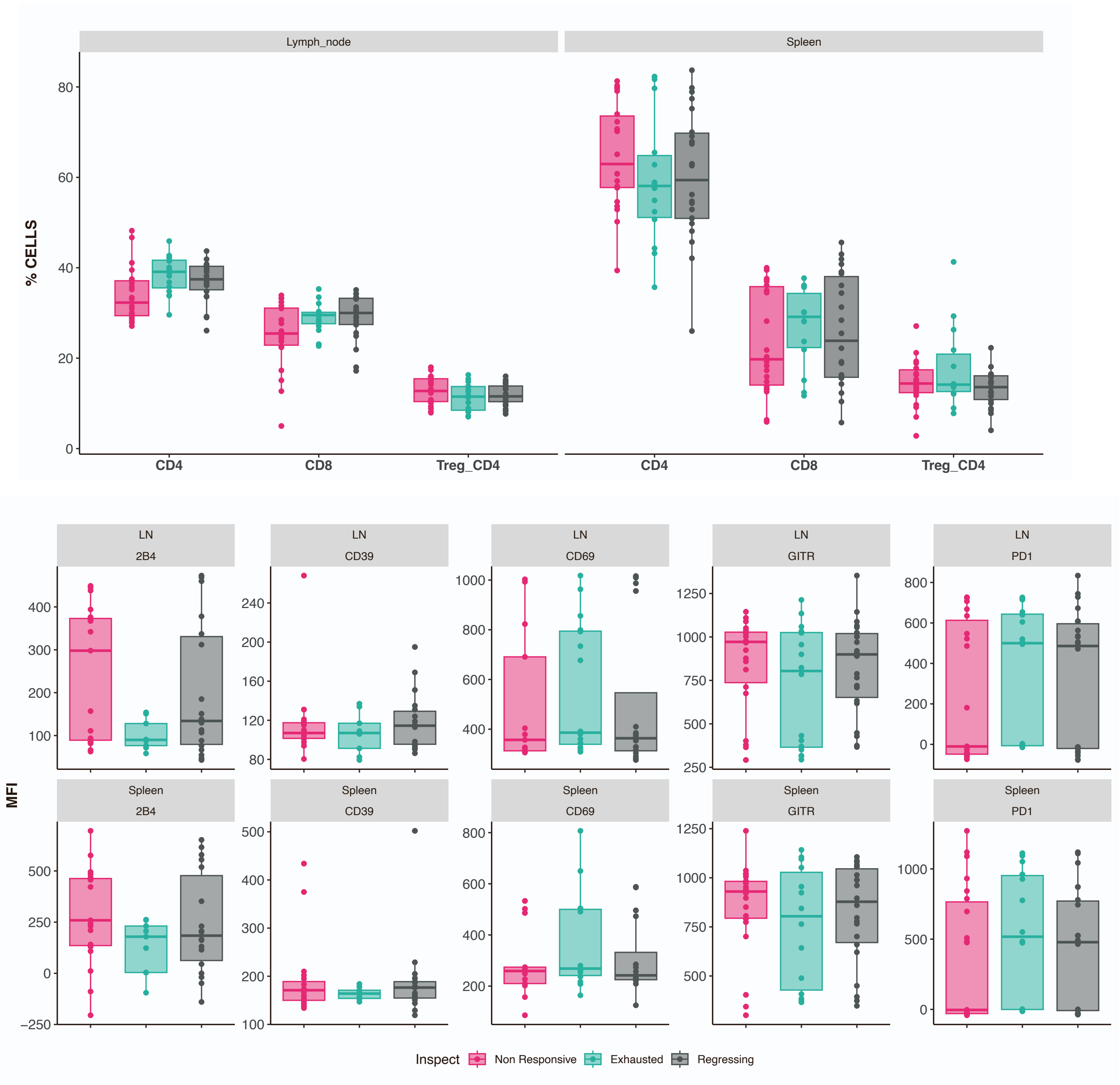

Lymph_node
Spleen
% CELLS
0
20
40
60
80
CD4
CD8
Treg_CD4
CD4
CD8
Treg_CD4
MFI
LN 2B4
LN CD39
LN CD69
LN GITR
LN PD1
Spleen 2B4
Spleen CD39
Spleen CD69
Spleen GITR
Spleen PD1
Inspect
Non Responsive
Exhausted
Regressing

Figure S5. Lymph node and spleen phenotyping of immune responses, related to Figure 4.

A. Total CD4 or CD8 T cells (as % of alive cells) or Tregs (as % of alive CD4 cells) in spleens and lymph nodes of tumour bearing mice separated by response. Symbols represent individual mice.
B. MFI of markers associated with exhaustion on CD8 of spleens and lymph nodes of tumour bearing mice separated by response. Symbols represent individual mice.

Figure S6

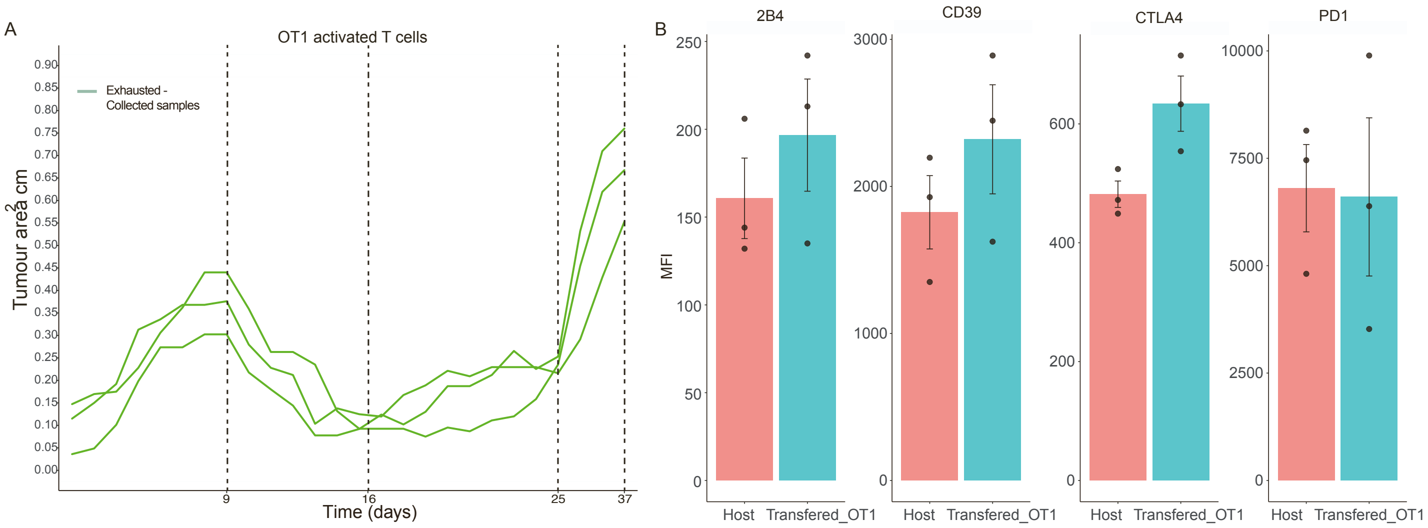

A
OT1 activated T cells
Exhausted -
Collected samples
Tumour area cm2
Time (days)
0.00
0.05
0.10
0.15
0.20
0.25
0.30
0.35
0.40
0.45
0.50
0.55
0.60
0.65
0.70
0.75
0.80
0.85
0.90
9
16
25
37
B
2B4
CD39
CTLA4
PD1
MFI
Host
Transfered_OT1

Figure S6. Tumour growth in cell transfer experiment, related to Figure 5.

A. Tumour growth curves of collected samples.

B. Expression of 2B4, CD39 and CTLA-4 of host and transferred CD8s TILs (identified by Vα2 TCR).

Figure S7

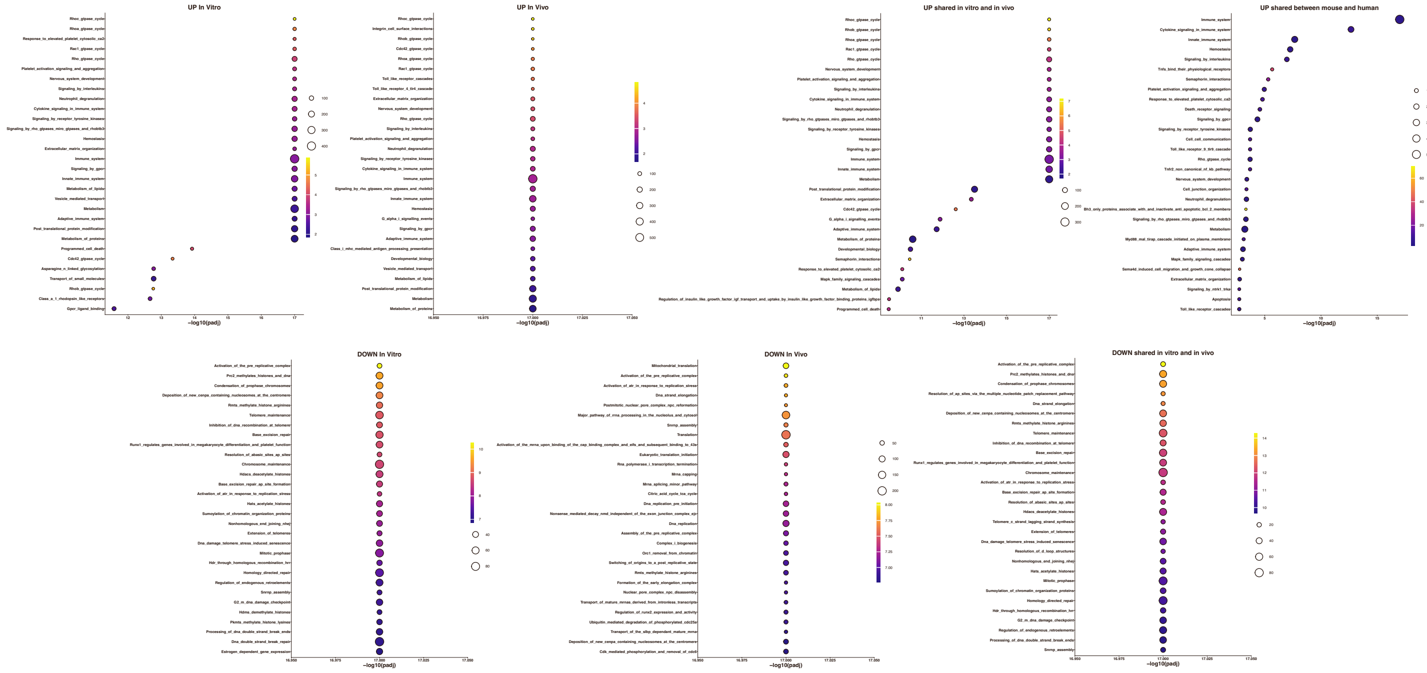

UP In Vitro
UP In Vivo
UP shared in vitro and in vivo
UP shared between mouse and human
DOWN In Vitro
DOWN In Vivo
DOWN shared in vitro and in vivo
-log10(padj)

Figure S7. Enriched pathways found in signatures, related to Figure 5.

Top 30 enriched pathways for each group from RNA-seq signatures gene sets. Bubble size reflects number of genes in pathways and colour gradient is fold enrichment.

Figure S8

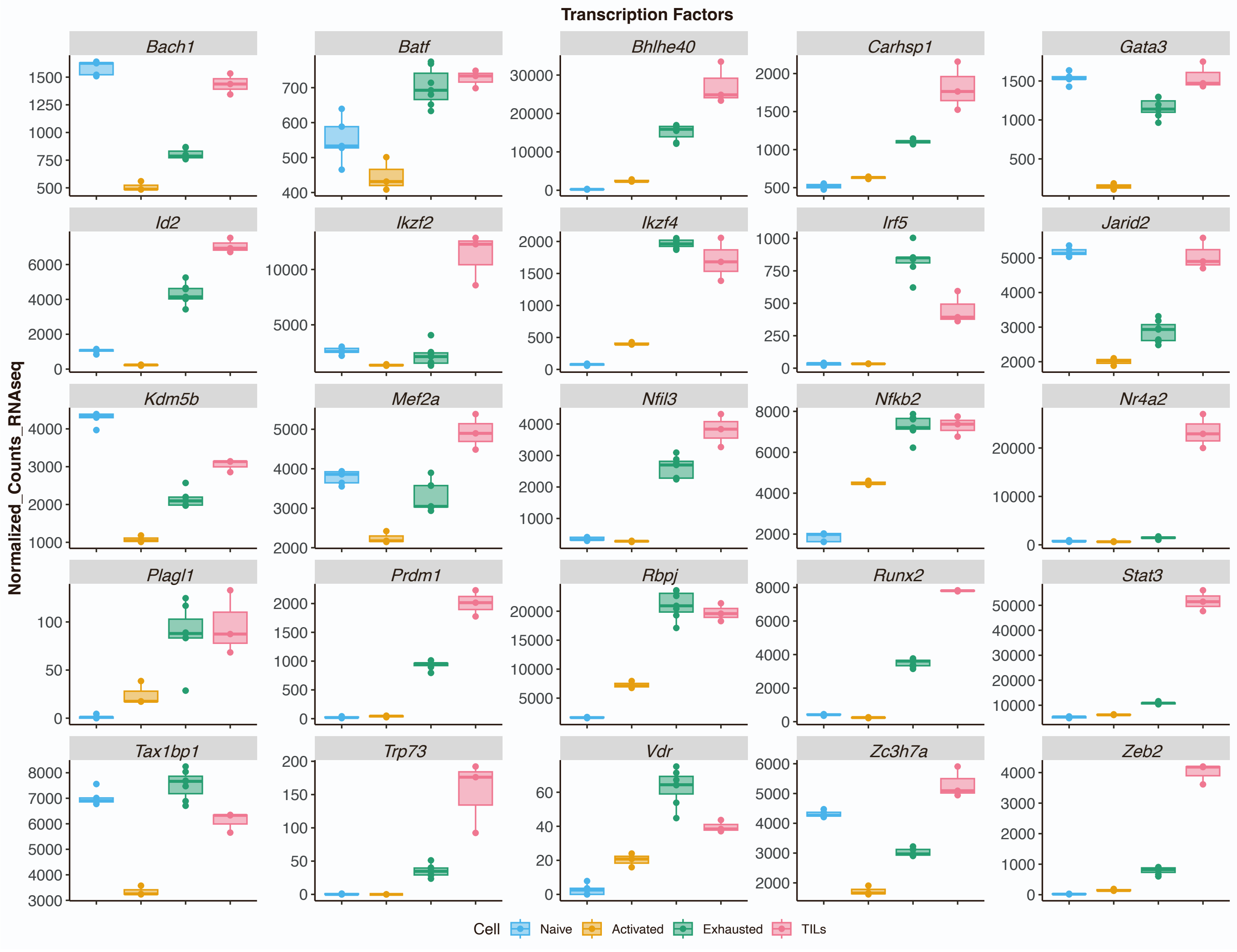
Transcription Factors
Bach1
Batf
Bhlhe40
Carhsp1
Gata3
Id2
Ikzf2
Ikzf4
Irf5
Jarid2
Kdm5b
Mef2a
Nfil3
Nfkb2
Nr4a2
Plagl1
Prdm1
Rbpj
Runx2
Stat3
Tax1bp1
Trp73
Vdr
Zc3h7a
Zeb2
Normalized_Counts_RNAseq
Cell
Naive
Activated
Exhausted
TILs

Figure S8. Shared upregulated transcription factors, related to Figure 5.

Log2 transformed normalised counts from naïve, activated and exhausted *in vitro* T cells, and *in vivo* CD8 TILs for the 25 shared up regulated transcription factors identified in figure 5C.

Figure S9

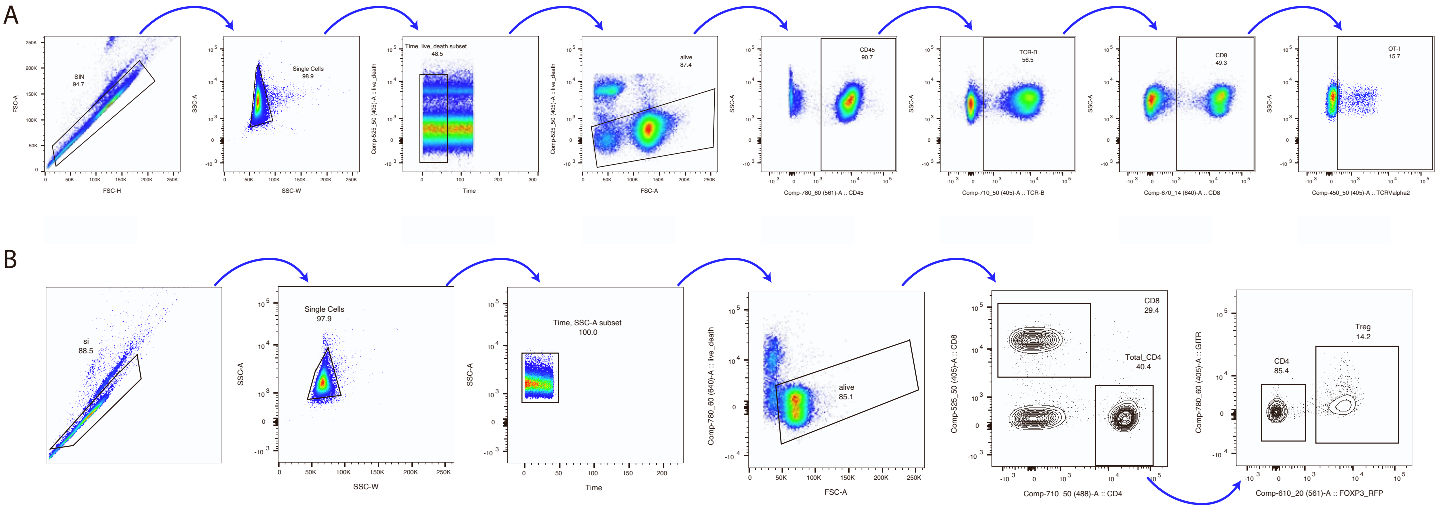
A
SIN
94.7
FSC-A
FSC-H
Single Cells
98.9
SSC-A
SSC-W
Time, live_death subset
48.5
Time
alive
87.4
FSC-A
CD45
90.7
Comp-780_60 (561)-A :: CD45
TCR-B
56.5
Comp-710_50 (405)-A :: TCR-B
CD8
49.3
Comp-670_14 (640)-A :: CD8
OT-I
15.7
Comp-450_50 (405)-A :: TCRValpha2
B
si
88.5
Single Cells
97.9
SSC-A
SSC-W
Time, SSC-A subset
100.0
Time
Comp-780_60 (640)-A :: live_death
alive
85.1
FSC-A
Comp-525_50 (405)-A :: CD8
CD8
29.4
Total_CD4
40.4
Comp-710_50 (488)-A :: CD4
Comp-780_60 (405)-A :: GITR
Treg
14.2
CD4
85.4
Comp-610_20 (561)-A :: FOXP3_RFP

Figure S9. Flow cytometry gating strategy for tissue samples, related to Figure 4,5.

A. Identification of CD8 and OT-I T cells from adoptively transferred mice.
B. Identification of CD8 and CD4 total, plus CD4 subsets (CD4 Foxp3- and CD4 Foxp3+) from mice.
